# Network meta‐analysis of rare events using penalized likelihood regression

**DOI:** 10.1002/sim.9562

**Published:** 2022-08-26

**Authors:** Theodoros Evrenoglou, Ian R. White, Sivem Afach, Dimitris Mavridis, Anna Chaimani

**Affiliations:** ^1^ Université Paris Cité, Research Center of Epidemiology and Statistics (CRESS‐U1153), INSERM Paris France; ^2^ MRC Clinical Trials Unit, University College London London UK; ^3^ Université Paris‐Est Créteil, UPEC Créteil France; ^4^ Department of Primary Education University of Ioannina Ioannina Greece; ^5^ Cochrane France Paris France

**Keywords:** bias reduction, maximum likelihood estimates, multiple treatment meta‐analysis, rare endpoints

## Abstract

Network meta‐analysis (NMA) of rare events has attracted little attention in the literature. Until recently, networks of interventions with rare events were analyzed using the inverse‐variance NMA approach. However, when events are rare the normal approximations made by this model can be poor and effect estimates are potentially biased. Other methods for the synthesis of such data are the recent extension of the Mantel‐Haenszel approach to NMA or the use of the noncentral hypergeometric distribution. In this article, we suggest a new common‐effect NMA approach that can be applied even in networks of interventions with extremely low or even zero number of events without requiring study exclusion or arbitrary imputations. Our method is based on the implementation of the penalized likelihood function proposed by Firth for bias reduction of the maximum likelihood estimate to the logistic expression of the NMA model. A limitation of our method is that heterogeneity cannot be taken into account as an additive parameter as in most meta‐analytical models. However, we account for heterogeneity by incorporating a multiplicative overdispersion term using a two‐stage approach. We show through simulation that our method performs consistently well across all tested scenarios and most often results in smaller bias than other available methods. We also illustrate the use of our method through two clinical examples. We conclude that our “penalized likelihood NMA” approach is promising for the analysis of binary outcomes with rare events especially for networks with very few studies per comparison and very low control group risks.

## 1 INTRODUCTION

Network meta‐analysis (NMA) has become an essential tool of comparative effectiveness research.^1^ Despite the rapid and extensive development of NMA methods over the last decade,^2^ little attention has been given to the issue of rare events within a network of interventions. Typically, NMAs with rare events are performed using the standard “inverse‐variance” (IV) model with a continuity correction for studies with zero events. This is a two‐stage contrast‐based model where at the first‐stage the effect sizes and their variances are extracted from the studies and at the second‐stage these are synthesized to obtain the summary treatment effect estimates. A major drawback of this approach is that it relies on large sample approximations and normality assumptions which are implausible under the presence of low number of observed events. An alternative popular approach, usually fitted in Bayesian framework, is based on the exact binomial distribution. However, it has been suggested that Bayesian methods may be problematic for meta‐analyzes of rare events since results can be dominated by the prior distribution.^3^, ^4^ On the other hand, when such a model is fitted in frequentist framework it relies on the maximum likelihood estimates (MLE) which are known to be biased in the presence of rare events.^5^, ^6^ Other, possibly less biased, options for performing NMA with rare events are the recent extension of the Mantel‐Haenszel (MH) meta‐analytical model^4^ and a model based on the noncentral hypergeometric distribution (NCH).^3^ The MH model, though, is a nonparametric common‐effect model and, thus, it avoids the use of any distributional assumption.

A further important issue of most meta‐analyzes with rare events is how to handle studies with zero events in all treatment groups. Imputations of arbitrarily chosen constants (eg, 0.5) have been found to bias the results.^7^, ^8^ More sophisticated models (eg, random intercepts models) allow inclusion of such studies by using between‐study information which, however, may again bias the results.^9^, ^10^ Existing methods for NMA with rare events that avoid the use of between‐study information, like the aforementioned MH‐NMA and NCH‐NMA models, either exclude these studies from the analysis or require continuity corrections. Studies with zero events in all treatment groups are not infrequent in meta‐analyzes of rare endpoints and the optimal way to treat such studies is still unclear. A recent meta‐epidemiological study of 442 Cochrane reviews including at least one study with no events in both treatment groups suggested that the inclusion or exclusion of these studies can impact materially the results of the meta‐analyses.^11^ In addition, in the context of NMA, exclusion of these studies may lead to disconnected networks.

In the present article, we aim to tackle the problem of rare events in networks of interventions with binary data by adapting and extending methodology from the analysis of individual studies. We use the logistic regression expression of NMA^12^ and the penalization to the likelihood function proposed by Firth^13^ to estimate the NMA relative effects, using odds ratios, through an one‐stage model. In this way, we attempt to likely obtain less biased and more precise estimates in comparison to the existing methods for NMAs with rare events described earlier. On top of that, our penalized likelihood NMA method (PL‐NMA) allows the inclusion of studies with zero events in all treatment groups without using between‐study information. The rest of the article is structured as follows. In Section 2, we describe the standard NMA model as a logistic regression model and the proposed PL‐NMA model. Section 3 presents a simulation study exploring the performance of our method in terms of bias and precision in comparison to existing NMA approaches for rare events under different scenarios. Finally, in Section 4 we apply the different models in two exemplar NMA datasets, and in Section 5 we discuss the strengths and limitations of our PL‐NMA approach.

## 2 METHODS

### 2.1 Common‐effect NMA using logistic regression

Suppose a network of N studies and T treatments. Let $r_{\mathrm{ik}}$ and $n_{\mathrm{ik}}$ denote the number of events and the number of total participants, respectively, in treatment group k of study i where i=1,2,…,N and $k\in K_{i}$ with $K_{i}=\left\{\text{treatments evaluated in study}\;i\right\}$. We assume that

$$r_{\mathrm{ik}}∼\mathrm{Bin}\left(n_{\mathrm{ik}},p_{\mathrm{ik}}\right),$$

where $p_{\mathrm{ik}}$ is the probability of an event in treatment group k of study i. Then, we model the probabilities $p_{\mathrm{ik}}\;$ through the following logistic regression model,

(1) $$\text{logit}\left(p_{\mathrm{ik}}\right)=\alpha_{i}+d_{b_{i}k}$$

with $b_{i}\;$ an arbitrarily chosen baseline treatment from the set $K_{i}$.

Parameter $d_{b_{i}k}$ represents the log‐odds ratio (logOR) of treatment k vs $b_{i}\;$ in the ith study with $d_{b_{i}b_{i}}=0$ for $k=b_{i}$. The parameter $a_{i}$ represents the log‐odds of the event in the $b_{i}\;$ group and it is treated as a nuisance parameter. Under the transitivity assumption, the logOR between any two treatments $t_{1}$ and $t_{2}$ ($t_{1},t_{2}=1,\ldots ,T$ and $t_{1}\neq t_{2}$) is

$$d_{t_{1}t_{2}}=d_{b_{i}\;t_{2}}-d_{b_{i}\;t_{1}}.$$

It follows from (1) that,

$$\;p_{\mathrm{ik}}=\text{expit}\left(\alpha_{i}+d_{b_{i}k}\right).$$

Let α denote the vector that contains all study intercepts $\alpha_{i}$, d the vector of the relative effects $d_{b_{i}k}$, and r and n the vectors of observed events and sample sizes per study and treatment arm.

The estimation of the parameters $d_{b_{i}k}$ relies on the maximization of the likelihood function which can be written as,

$$L\left(\alpha ,d\;|\;r,n\right)=\prod_{i=1}^{N}\prod_{k\in K_{i}}\left(\begin{array}{c} n_{\mathrm{ik}}\\ r_{\mathrm{ik}} \end{array}\right)\text{expit}{\left(\alpha_{i}+d_{b_{i}k}\right)}^{r_{\mathrm{ik}}}\;{\left(1-\text{expit}\left(\alpha_{i}+d_{b_{i}k}\right)\right)}^{n_{\mathrm{ik}}-r_{\mathrm{ik}}}.$$

Equivalently, the log‐likelihood function is,

(2) $$l\left(\alpha ,d\;|\;r,n\right)=\sum_{i=1}^{N}\sum_{k\in K_{i}}\log\left(\begin{array}{c} n_{\mathrm{ik}}\\ r_{\mathrm{ik}} \end{array}\right)+r_{\mathrm{ik}}\log\left(\text{expit}\left(\alpha_{i}+d_{b_{i}k}\right)\right)+\left(n_{\mathrm{ik}}-r_{\mathrm{ik}}\right)\log\left(1-\text{expit}\left(\alpha_{i}+d_{b_{i}k}\right)\right).$$

The above model relies on the maximization of the log‐likelihood in Equation (2) in terms of the N+T−1 parameters a and d. These MLE are biased in the case of finite sample sizes and their unbiasedness can only be assumed approximately for a large sample size and a considerable number of observed events.^5^ Hence, the MLE can be problematic when the number of observed events is small, underestimating the probability of an event and overestimating the treatment effects.^14^, ^15^

### 2.2 Common‐effect penalized likelihood NMA

To reduce the bias of the above MLE for NMAs with rare events, we employ Firth's modification^13^ to the log‐likelihood function in (2); this is the frequentist equivalent to using as penalty Jeffrey's invariant prior. This modification results in the penalized likelihood and log‐likelihood functions, respectively,

$$L^{*}\left(\alpha ,d|r,n\right)=L\left(\alpha ,d|r,n\right)\;{\left|I\right|}^{\frac{1}{2}}$$

and

(3) $$l^{*}\left(\alpha ,d|r,n\right)=l\left(\alpha ,d|r,n\right)+\frac{1}{2}\log\left|I\right|.$$

The matrix I≡I(α,d) is the Fisher's information matrix with dimensions (N+T−1)×(N+T−1) that is given as

(4) $$I=Z^{\prime }\mathrm{WZ}$$

with Z being the model's design matrix with dimensions $\sum_{i=1}^{N}A_{i}\times \left(N+T-1\right)$ and entries 1 for the columns associated with the relevant treatment and study and 0 otherwise.^16^ $A_{i}\;$ is the number of arms in study i, $W=\mathrm{diag}\{\;n_{\mathrm{ik}}p_{\mathrm{ik}}(1-p_{\mathrm{ik}})\}$ and |I| is the determinant of I. The penalized likelihood function is being maximized with respect to the (N+T−1) parameters a and d.

Equation (3) arises by the Taylor series expansion of the score function (ie, derivative of the log‐likelihood function). Firth showed that the use of Jeffrey's invariant prior in this expansion penalizes the likelihood function and provides less biased estimates than the standard likelihood^13^ function of Equation (2). The estimates obtained from the penalized likelihood function are typically shrunk toward 0 and this in turn results in smaller SEs. This is why the estimates obtained from the penalized likelihood function are expected to be more precise than those obtained from the standard likelihood function.^17^ The above PL‐NMA provides finite treatment effects and SEs even in the case of studies that report zero events in all treatment arms.^17^, ^18^ Specifically, the issue of studies reporting only zero events in meta‐analysis resembles that of separation (ie, when one or more covariates perfectly predict the outcome) in individual studies.^19^, ^20^ The PL‐NMA method allows the inclusion of studies with zero events in two or more groups, without sharing information between the studies, by adding some prior information to the probability of an event through Jeffrey's prior. The latter usually results in estimated probabilities of event which are shifted toward 0.5.^17^, ^20^

After maximizing the penalized likelihood function, we can replace $p_{\mathrm{ik}}$ with ${\hat{p}}_{\mathrm{ik}}$ in matrix W. Those probabilities can be easily obtained as ${\hat{p}}_{\mathrm{ik}}=\text{expit}\left({\hat{a}}_{i}+{\hat{d}}_{b_{i}k}\right).\;$ The square roots of the diagonal elements of the $I^{-1}$ represent the SEs of the estimates which are used to construct Wald type confidence intervals; for instance, the square root of the diagonal element in the first row and first column of matrix $I^{-1}$ represents the SE of the parameter in the first column of matrix Z. The matrix $I^{-1}$ is calculated as the inverse of matrix I in Equation (4). An alternative option is to construct profile likelihood confidence intervals that can be obtained using the critical region defined by the inequality

$$2\left\{l^{*}\left(\hat{\alpha },\hat{d}|r,n\right)-l^{*}\left(\hat{\alpha },{\hat{d}}_{0}|r,n\right)\right\}\leq c_{1,1-q},$$

where ${\hat{d}}_{0}=\left(d_{b_{i}k_{0}},{\hat{d}}_{b_{i}k}\right)\;$ is the vector that contains the estimated treatment effects of all treatments in the network vs the reference, except $k_{0}\neq k$ which is a specific treatment. The boundary $c_{1,1-q}$ is the (1−q) quantile of the chi‐square distribution with 1 degree of freedom.

In case of T=2, the model in (1) with the likelihood function in (3) corresponds to the penalized likelihood regression model for pairwise meta‐analysis.

### 2.3 Random‐effects penalized likelihood NMA

The model in Equation (1) can be extended to the so‐called binomial‐normal (BN‐NMA) model if we replace the $d_{b_{i}k}$ with $\delta_{i,b_{i}k}$ which are now the study‐specific treatment effects that are assumed to follow a normal distribution with mean $d_{b_{i}k}\;$ and variance $\tau^{2}$. In this model, the true treatment effects vary from study to study but the study intercepts $\alpha_{i}$ are kept fixed. The model can also allow for random intercept terms. The random‐intercepts model requires a common reference treatment across the studies.^21^ This theoretical common reference does not have to be observed within each study but its impact needs to be independent from unmeasured covariates. The PL‐NMA model is a common‐effect model and its extension to incorporate heterogeneity is challenging. In particular, the standard approach of incorporating heterogeneity as an additive term is not straightforward as the penalization requires closed forms of the likelihood function and its moments^13^ and these are not available for a logistic regression mixed‐effect model. Therefore, we use an alternative approach previously suggested in the literature where heterogeneity is incorporated as a multiplicative term.^22^, ^23^, ^24^ This is a two‐stage approach that modifies the study variances through an overdispersion parameter. Specifically, after fitting the model presented in the previous section, we multiply the variance in study i and arm $k\;v_{\mathrm{ik}}=n_{\mathrm{ik}}p_{\mathrm{ik}}\left(1-p_{\mathrm{ik}}\right)$ with a scale parameter φ, ^21^, ^22^, ^23^ hence

$$v_{\mathrm{ik}}^{*}=v_{\mathrm{ik}}*\varphi ,\;\varphi \geq 1.$$

This implies that the matrix W in Equation (4) is replaced by φW. In this way, the point estimates of the treatment effects remain unaffected but their variances are inflated by that unknown parameter φ. To estimate the parameter φ we use the expression suggested by Fletcher^25^

$$\hat{\varphi }=\frac{{\hat{\varphi }}_{P}}{1+\bar{s}},$$

where $\bar{s}\;$ is the mean value of $s_{\mathrm{ik}}=\left(\frac{\partial {\hat{v}}_{\mathrm{ik}}}{\partial {\hat{p}}_{\mathrm{ik}}}\right)\frac{\left(r_{\mathrm{ik}}-\hat{E}\left(r_{\mathrm{ik}}\right)\right)}{{\hat{v}}_{\mathrm{ik}}}$, $\frac{\partial {\hat{v}}_{\mathrm{ik}}}{\partial {\hat{p}}_{\mathrm{ik}}}$ is the derivative of ${\hat{v}}_{\mathrm{ik}}$ with respect to ${\hat{p}}_{\mathrm{ik}}$ and ${\hat{\varphi }}_{P}=\frac{P}{m}$ with P denoting the Pearson's statistic, and m=(T−1)+(N−1) the residual degrees of freedom of the model. The Pearson's statistic for the NMA model presented in the previous section is,

$$P=\sum_{i=1}^{N}\sum_{k\in K_{i}}\frac{r_{\mathrm{ik}}-\hat{E}\left(r_{\mathrm{ik}}\right)}{{\hat{v}}_{\mathrm{ik}}},$$

where $\hat{E}\left(r_{\mathrm{ik}}\right)=n_{\mathrm{ik}}*{\hat{p}}_{\mathrm{ik}}.\;$ If $\hat{\varphi }<1$, we set it equal to 1.

## 3 SIMULATION

We conducted a simulation study to compare our proposed and existing methodologies for NMA of rare events. We report our simulation following the recommendations by Morris et al^26^

### 3.1 Data generation

We start the data generation process by specifying the number of treatments in the network (3, 5, or 8) and the number of studies in every treatment comparison (2, 4, or 8). We always create all possible $\frac{T\left(T-1\right)}{2}$ treatment comparisons; hence we only assume fully‐connected networks. We then generate the total number of participants per study arm using a uniform distribution, namely, $n_{\mathrm{ik}}∼\text{Unif}\left(c_{1},c_{2}\right)$, with $c_{1}=30,c_{2}=60$ for generating small studies and $c_{1}=100,c_{2}=200$ for larger studies. We only generated studies with an equal number of participants across arms. For each study we generated the control group risk; that is the risk of the event for patients receiving the reference treatment irrespective of whether this was evaluated in the study, thus for the simulation $b_{i}\equiv 1$. We generate the risk of an event in the reference treatment group 1 using $p_{i1}∼\text{Unif}\left(u_{1},u_{2}\right),$ with $u_{1}\in \left[0.5\%-5\%\right],u_{2}\in \left[1\%-10\%\right]$ depending on the scenario (see Table 1). In all scenarios, the true logORs were fixed at equal intervals between 0 and 1 (eg, in a network of 5 treatments the true logORs are set to 0.25, 0.5, 0.75, and 1 for the comparisons of treatments 2, 3, 4, and 5 vs the reference treatment 1, respectively) and were used to calculate the odds of an event in every study arm,

$${\text{odds}}_{i1}=\frac{p_{i1}}{1-p_{i1}},$$

$${\text{odds}}_{\mathrm{ik}}={\text{odds}}_{i1}*\text{OR},k\neq 1.$$

**TABLE 1:** Overview of scenarios examined in our simulation. For each scenario we generated 1000 datasets. The rows in bold represent the scenarios with multi‐arm studies

#	Treatments in the network	Patients per arm	Number of studies per comparison	Total number of studies per dataset	Heterogeneity (τ)	Control group risk (%)	Mean events per study
1	5	30–60	2	20	0	3%–5%	3
2	5	30–60	2	20	0.1	3%–5%	3
3	5	30–60	2	20	0	5%–10%	6
4	5	30–60	2	20	0.1	5%–10%	6
5	5	30–60	4	40	0	3%–5%	3
6	5	30–60	4	40	0.1	3%–5%	3
7	5	30–60	4	40	0	5%–10%	6
8	5	30–60	4	40	0.1	5%–10%	6
9	8	30–60	2	56	0	3%–5%	3
10	8	30–60	2	56	0.1	3%–5%	3
11	5	100‐200	2	20	0	1%–2%	4
12	5	100–200	2	20	0.1	1%–2%	4
13	5	100–200	2	20	0	0.5%–1%	2
14	5	100–200	2	20	0.1	0.5%–1%	2
15	5	100–200	4	40	0	0.5%–1%	2
16	5	100–200	4	40	0.1	0.5%–1%	2
**17**	**3**	**100–200**	**8**	**8**	**0**	**1**%**–2%**	**4**
**18**	**3**	**100–200**	**8**	**8**	**0.1**	**1**%**–2%**	**4**
**19**	**3**	**100–200**	**8**	**8**	**0**	**0.5**%**–1%**	**2**
**20**	**3**	**100–200**	**8**	**8**	**0.1**	**0.5**%**–1%**	**2**
**21**	**3**	**100–200**	**8**	**8**	**0**	**0.5**%**–5%**	**7**
**22**	**3**	**100–200**	**8**	**8**	**0.1**	**0.5%–5%**	**7**
**23**	**3**	**100–200**	**8**	**8**	**0**	**0.5%–10%**	**13**
**24**	**3**	**100–200**	**8**	**8**	**0.1**	**0.5%–10%**	**13**
**25**	**5**	**100–200**	**8**	**8**	**0**	**1%–2%**	**4**
**26**	**5**	**100–200**	**8**	**8**	**0.1**	**1%–2%**	**4**
**27**	**5**	**100–200**	**8**	**8**	**0**	**0.5%–1%**	**2**
**28**	**5**	**100–200**	**8**	**8**	**0.1**	**0.5%–1%**	**2**
**29**	**5**	**100–200**	**8**	**8**	**0**	**0.5–5%**	**7**
**30**	**5**	**100–200**	**8**	**8**	**0.1**	**0.5%–5%**	**7**
**31**	**5**	**100–200**	**8**	**8**	**0**	**0.5%–10%**	**13**
**32**	**5**	**100–200**	**8**	**8**	**0.1**	**0.5%–10%**	**13**
33	5	100–200	4	40	0	0.1%–0.3%	1

Finally, we obtain the event probabilities in the non‐reference treatment groups as $p_{\mathrm{ik}}=\frac{{\text{odds}}_{\mathrm{ik}}}{1+{\text{odds}}_{\mathrm{ik}}}$ and the number of events in every study arm using a binomial distribution $r_{\mathrm{ik}}∼\mathrm{Bin}\left(n_{\mathrm{ik}},p_{\mathrm{ik}}\right),\forall i,k$.

For scenarios assuming the presence of heterogeneity (τ=0.1), we set a common parameter τ to represent the SD of the random‐effects (see the Supplementary material S1 for a description).

We explored in total 33 scenarios with varying control group risks in the studies, number of treatments in the network, study size, number of studies per comparison, and magnitude of heterogeneity (Table 1). In scenarios 1–16 we considered only two‐arm studies while in scenarios 17–32 we only included multi‐arm studies evaluating all treatments. The latter are certainly not very realistic scenarios but they aimed at exploring whether the increased correlation from multi‐arm studies could contribute to precision and bias reduction in NMAs with rare events. In scenario 33, we set extremely low control group risks (Table 1) to increase the number of generated studies with zero events in all treatment arms. This scenario only included two‐arm studies.

We considered both homogeneous and heterogeneous cases and with different number of studies per comparison. Scenarios 1–10 only considered small studies (ie, 30–60 participants per arm). For scenarios 11–32, we increased the number of patients per arm and we mostly focus on cases in which the control group risks were extremely low (ie, 1%–2% and 0.5%–1%). In Scenarios 21–24 and 29–32 we considered a mix of low with higher control group risks. Table 1 provides a summary of the different scenarios. Across the first 32 scenarios the majority of the 1000 simulated datasets included only a handful of studies reporting only zero events. Therefore, in the final scenario 33 we further decreased the control group risk to lie between 0.1% and 0.3% in order to generate more studies with zero events in all treatment groups. Overall, though, the impact of such studies on the final results cannot be fully captured through the present simulation study and further work is necessary. The extent of all‐zero event studies can be found in Table 3 of our Supplementary material S1.

### 3.2 Evaluated models

The simulation was conducted using R version 4.0.3 (October 10, 2020) and the packages netmeta,^27^ brglm,^28^ lme4,^29^ and gemtc.^30^ The packages netmeta^27^ and gemtc^30^ are tailored for frequentist and Bayesian NMA, respectively. The packages lme4^29^ and brglm^28^ allow the conduction of NMA either as a generalized linear mixed effects model or as a generalized linear model, respectively. The code used for our simulation can be found at https://github.com/TEvrenoglou/PL‐NMA. In each scenario, we generated 1000 datasets and we compared the following models:

- Common‐ and random‐effects IV‐NMA model with 0.5 correction for studies with at least one zero event arm
- Common‐effect MH‐NMA and NCH‐NMA without 0.5 correction for studies with zero event arms. Note that the NCH‐NMA model also allows for random effects. However, the version of this model used here is the one implemented in netmeta^27^ which is only common‐effect and uses Breslow's approximation.
- Common‐ and random‐effects PL‐NMA model.
- Common‐effect logistic regression NMA model with standard (non‐penalized) likelihood.
- Random‐effects logistic regression NMA model using MLE with fixed study intercept (BN‐NMA). The model assumes the exact binomial likelihood for the observed events within studies and a normal distribution for the random‐effects across the studies. The glmer function from the lme4^29^ package was used to perform NMA. This function is not tailored for NMA and does not meet the requirement that variance‐covariance matrix of the random‐effects is having the structure with $\tau^{2}$ for diagonal elements and $\frac{\tau^{2}}{2}$ for off‐diagonal elements. The latter implies that this function can only be used for scenarios 1–16 where NMA of only two arm studies takes place.
- Common and random‐effects Bayesian NMA with exact binomial likelihood and non‐informative priors for the treatment effects. The common‐effect model assumes that $d_{b_{i}k}∼N\left(0,100^{2}\right)$. For random‐effects the study‐specific treatment effects are following a normal distribution $\delta_{i,b_{i}k}∼N(d_{b_{i}k},\tau^{2})$, then we form two random‐effects models: the first assumes that $d_{b_{i}k}∼N({0,100}^{2})$ and for the heterogeneity parameter that τ∼Unif(0,2). ^31^ The second random‐effects model assumes narrower priors; $d_{b_{i}k}∼N(0,10^{2})$ and a half‐normal distribution for τ∼HN(1). For all Bayesian models, we run 2 chains with 50 000 iterations and discarded the first 10 000 samples from each chain. Convergence was checked using the Brooks–Gelman–Rubin criterion^32^ with a value lower than 1.1 considered as indicating convergence.

The objective of scenario 33 was to investigate the impact of all‐zero event studies. Therefore, in this scenario we excluded the IV‐NMA, the MH‐NMA, and the NCH‐NMA models as those models need to exclude all‐zero event studies prior to the analysis.^4^ The rest of the models were evaluated once by including and once by excluding all‐zero event studies. A brief description of all models that are not described in the article is provided in the Supplementary material S1.

### 3.3 Estimands and performance measures

The estimands of interest are the T−1 logORs between treatments t=2,…,T and treatment 1. The performance of the models was investigated in terms of the mean bias defined as the mean difference between the estimated and the true logORs averaged over simulated datasets and estimands for each NMA method: the true estimands are all positive so averaging over estimands makes sense. For heterogeneity parameter in terms of the models assuming additive heterogeneity terms we calculated the mean difference between the estimated and the true τ averaged over simulated datasets while for the multiplicative PL‐NMA model we report the percentage of the simulated datasets for which $\hat{\varphi }>1.$ We further calculated the mean coverage in each scenario defined as the percent of the corresponding 95% confidence or credible intervals that included the corresponding true logOR. Finally, the methods were compared in terms of the mean squared error (MSE) and length of confidence or credible intervals averaged across multiple contrasts and simulated datasets.

### 3.4 Simulation results

In the following sections we present the results of our simulation across the different performance measures. We first discuss the results for scenarios 1–32 and then we discuss separately the results of scenario 33. Overall, the Bayesian random‐effects model with the uniform prior for the heterogeneity parameter had a rather poor performance and therefore its results are not discussed in the text but only presented in the figures and the tables; the results of all other models are discussed in the following sections.

#### 3.4.1 Bias in the estimation of the logORs

**FIGURE 1:**
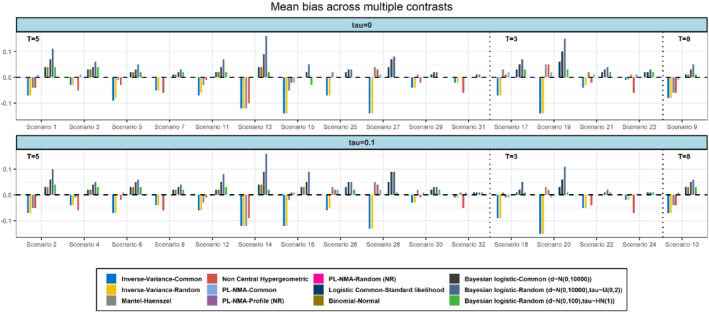
Simulation results in terms of mean bias for scenarios with 5, 3, and 8 treatments (T), respectively. Missing bars correspond to 0 mean bias (after rounding to two decimal places) for the respective NMA method. The Monte‐Carlo standard error across the different scenarios and methods ranges from 0.004 to 0.02 with a mean value equal to 0.01. Models marked as *NR* are not relevant to the figure and thus no results are plotted

In Figure 1, we present the results in terms of mean bias across multiple contrasts (multiple estimands). In most scenarios, IV‐common‐effect and IV‐random‐effects gave the most biased results. The two PL‐NMA models overall produced the least biased results across scenarios. The MH‐NMA, NCH‐NMA, and BN‐NMA models also performed generally well, but the MH‐NMA and NCH‐NMA resulted in large bias in scenarios with many treatments in the network and a small number of studies per comparison (ie, scenarios 13, 14). The performance of NCH model for the scenarios with higher risks (scenarios 3, 4, 7, 8, 23, 24, 31, 32) was decreased as this model uses Breslow's approximation which is valid only for very rare events (see the Supplementary material S1 for details). Interestingly, the PL‐NMA models had a stable performance across the different scenarios in terms of bias with a maximum value of mean bias equal to 0.02.

The BN‐NMA model provided satisfactory results in terms of bias in almost all scenarios with a maximum mean bias of 0.06. The performance of BN‐NMA was increased in scenarios involving relatively larger studies, namely, when the number of participants per arm was between 100 and 200. The results of the common‐effect logistic regression model with the standard (non‐penalized) likelihood were equivalent to the results of the BN‐NMA model. The random‐effects Bayesian model with half‐normal prior distribution for heterogeneity had an improved performance in terms of bias and a better performance in comparison to the common‐effect Bayesian model with bias ranging from −0.03 to 0.04. In all scenarios, the results in terms of bias were not affected materially for any of the models when the data‐mechanism involved heterogeneity. Overall, the IV‐NMA, the MH‐NMA, and the NCH‐NMA models had the tendency to give negative bias whether the rest of the models were positively biased. Full results are available in the Supplementary Tables 1 and 2 and are consistent with the results that we obtained for each contrast separately (Supplementary Figures 1–4).

#### 3.4.2 Estimation of heterogeneity parameter

In terms of estimation of the heterogeneity, all the random‐effects models appeared to have a bad performance (Table 2). The IV random‐effects model seemed to have a particularly poor performance for estimating $\hat{\tau }\neq 0$. Especially for scenario 16, where 3 treatments and 8 studies per comparison were available, the model estimated that $\hat{\tau }=0$ across 996 out of 1000 simulated datasets while the true value was set equal to 0.1. The BN‐NMA model had also a very poor performance in estimating τ as in all heterogeneous scenarios the heterogeneity parameter was estimated being 0. Finally, since there is no clear correspondence between τ and φ we calculated the percentage of times with $\hat{\varphi }>1\;$ across all datasets and scenarios. Across scenarios assuming heterogeneity (τ=0.1), $\hat{\varphi }$ was greater than 1 in a range between 0%–40% across the simulated datasets and scenarios; this percentage was increased to 20%–40% in scenarios with higher control group risks (scenarios 4, 8, 22, 24, 30, 32). Across scenarios assuming that τ=0 the percentage that $\hat{\varphi }>1$ ranges from 0‐36%. The percentage per scenario can be found in Table 2. The half‐normal Bayesian random‐effects model appeared to provide the most biased estimates of the heterogeneity parameter with bias ranging from 0.07 to 0.25.

**TABLE 2:** Results in terms of bias for the estimation of heterogeneity across all scenarios. For the case of PL‐NMA model the frequency that $\hat{\varphi }>1\;$ is reported instead of the bias. In parenthesis the percentage that $\hat{\tau }>0\;$ or $\hat{\varphi }>1$ across the 1000 datasets and 32 scenarios. In bold the scenarios that assume τ=0.1

#	IV‐random	Binomial normal	Bayesian $d∼N\left(0,100^{2}\right),$ τ∼U(0,2)	Bayesian $d∼N\left(0,10^{2}\right),$ τ∼HN(1)	PL‐NMA‐random
	Mean bias (% $\hat{\tau }\;$>0)	Mean bias (% $\hat{\tau }\;$>0)	Mean bias (% $\hat{\tau }\;$>0)	Mean bias (% $\hat{\tau }\;$>0)	Frequency $\hat{\varphi }>1\;$(% $\hat{\varphi }>1$)
1	0.05 (15.5%)	0 (0%)	0.54 (100%)	0.25 (100%)	94 (9.4%)
**2**	**−0.04 (16.5%)**	**−0.1 (0%)**	**0.46 (100%)**	**0.15 (100%)**	**101 (10.1%)**
3	0.09 (29.6%)	0 (0%)	0.36 (100%)	0.22 (100%)	246 (24.6%)
**4**	**0.01 (37%)**	**−0.1 (0%)**	**0.28 (100%)**	**0.13 (100%)**	**290 (29%)**
5	0.01 (5.5%)	0 (0%)	0.37 (100%)	0.23 (100%)	21 (2.1%)
**6**	**−0.08 (7.5%)**	**−0.1 (0%)**	**0.29 (100%)**	**0.13 (100%)**	**33 (3.3%)**
7	0.05 (22.8%)	0 (0%)	0.26 (100%)	0.20 (100%)	155 (15.5%)
**8**	**−0.03 (29.9%)**	**−0.1 (0%)**	**0.18 (100%)**	**0.11 (100%)**	**202 (20.2%)**
9	0.01 (3.3%)	0 (0%)	0.34 (100%)	0.23 (100%)	10 (1%)
**10**	**−0.09 (5.6%)**	**−0.1 (0%)**	**0.25 (100%)**	**0.13 (100%)**	**19 (1.9%)**
11	0.05 (17%)	0 (0%)	0.44 (100%)	0.23 (100%)	112 (11.2%)
**12**	**−0.03 (21.7%)**	**−0.1 (0%)**	**0.36 (100%)**	**0.14 (100%)**	**141 (14.1%)**
13	0.01 (2.6%)	0 (0%)	0.71 (100%)	0.25 (100%)	7 (0.7%)
**14**	**−0.09 (3.9%)**	**−0.1 (0%)**	**0.62 (100%)**	**0.16 (100%)**	**12 (1.2%)**
15	0.00 (0.3%)	0 (0%)	0.50 (100%)	0.24 (100%)	0 (0%)
**16**	**−0.10 (0.4%)**	**−0.1 (0%)**	**0.41 (100%)**	**0.14 (100%)**	**0 (0%)**
17	0.06 (18.4%)	‐	0.43 (100%)	0.23 (100%)	127 (12.7%)
**18**	**−0.03 (24.2%)**	‐	**0.34 (100%)**	**0.14 (100%)**	**161 (16.1%)**
19	0.01 (4.8%)	‐	0.65 (100%)	0.25 (100%)	18 (1.8%)
**20**	**−0.08 (5.1%)**	‐	**0.55 (100%)**	**0.15 (100%)**	**30 (3%)**
21	0.07 (27.8%)	‐	0.32 (100%)	0.21 (100%)	201 (20.1%)
**22**	**−0.01 (33.1%)**	‐	**0.23 (100%)**	**0.11 (100%)**	**236 (23.6%)**
23	0.08 (38.2%)	‐	0.24 (100%)	0.18 (100%)	321 (32.1%)
**24**	**−0.01 (41.9%)**	‐	**0.15 (100%)**	**0.09 (100%)**	**356 (35.6%)**
25	0.03 (14.8%)	‐	0.31 (100%)	0.21 (100%)	92 (9.2%)
**26**	**−0.06 (18.1%)**	‐	**0.22 (100%)**	**0.12 (100%)**	**120 (12%)**
27	0.00 (1.7%)	‐	0.47 (100%)	0.24 (100%)	11 (1.1%)
**28**	**−0.09 (2.3%)**	‐	**0.37 (100%)**	**0.14 (100%)**	**10 (1%)**
29	0.05 (25.3%)	‐	0.23 (100%)	0.18 (100%)	167 (16.7%)
**30**	**−0.03 (32.9%)**	‐	**0.15 (100%)**	**0.09 (100%)**	**238 (23.8%)**
31	0.06 (34.8%)	‐	0.17 (100%)	0.15 (100%)	276 (27.6%)
**32**	**−0.01 (47.6%)**	‐	**0.09 (100%)**	**0.07 (100%)**	**387 (38.7%)**

#### 3.4.3 Coverage probability

**FIGURE 2:**
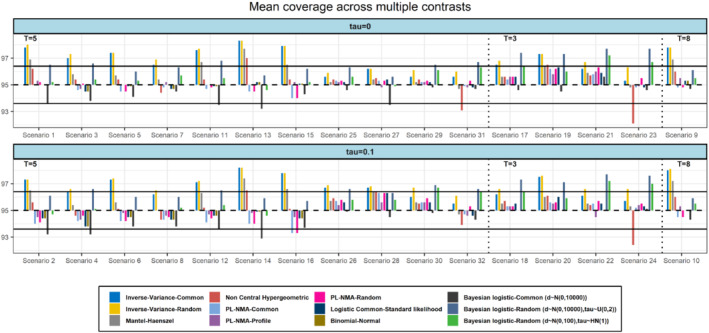
Simulation results in terms of coverage probability for scenarios with 5, 3, and 8 treatments (T), respectively. The horizontal lines represent the upper and lower bounds of the 95% confidence interval which is constructed for the nominal level

In terms of coverage probability, the PL‐NMA model with respect to Wald‐type confidence interval had a consistent performance with coverage ranging from 93.3% to 96.3% in 18 scenarios the coverage was slightly below the nominal level but almost always within 95% confidence interval constructed for the nominal level (Figure 2). This confidence interval was calculated as $0.95\pm 1.96\sqrt{\frac{0.95\left(1-0.95\right)}{1000}}=\left(0.936,0.964\right)\times 100\%$ . The performance of the PL‐NMA model was increased by using profile likelihood confidence intervals and, in most scenarios, it was around the nominal level of 95% with a range between 94.3% and 96%. MH‐NMA and NCH‐NMA models had similarly good performance with coverage probability close to the nominal level, but it should be noted that the performance of NCH‐NMA model was again decreased for some scenarios with higher control group risks (scenarios 23, 24, 31). These results are consistent with previous simulation studies.^4^ The IV‐NMA models had the worst performance as those models were suffering from over‐coverage across almost all scenarios. Overall, for IV‐NMA, MH‐NMA, and NCH‐NMA results in terms of coverage are consistent with previous simulation studies.^4^ The BN‐NMA model and two Bayesian models had in most scenarios a good performance with a coverage probability around the nominal level and typically within confidence interval bounds for the nominal level.

#### 3.4.4 MSE and length of confidence or credible intervals

**FIGURE 3:**
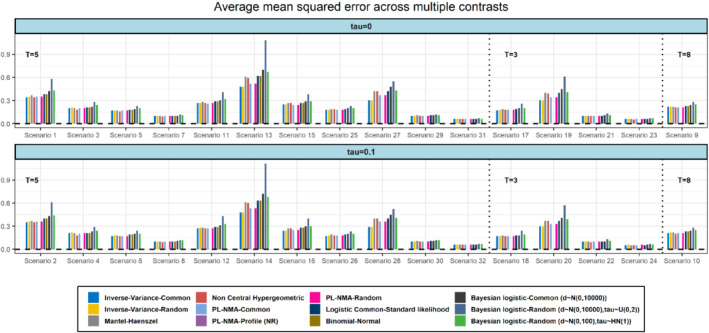
Simulation results in terms of MSE for scenarios with 5, 3, and 8 treatments (T), respectively. Models marked as *NR* are not relevant to the figure and thus no results are plotted

**FIGURE 4:**
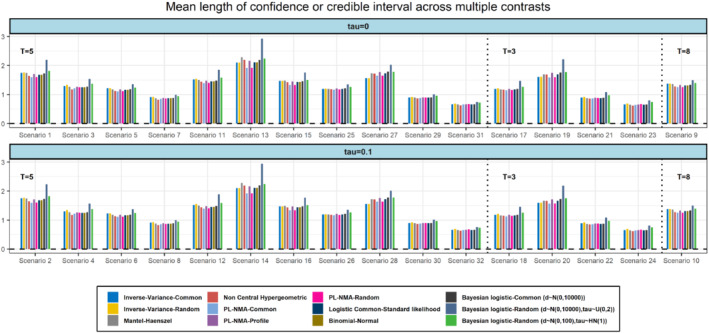
Simulation results in terms of length of confidence (or credible) intervals for scenarios with 5, 3, and 8 treatments (T), respectively

In terms of MSE all the models appeared to have a similar performance (Figure 3). The two Bayesian models had a slightly increased MSE which overall found to range from 0.07 to 0.72. Regarding the mean length of confidence (or credible intervals) the Wald‐type confidence intervals obtained by the PL‐NMA were found to have the smaller lengths in comparison to the other models (Figure 4). The mean length of the profile‐likelihood confidence intervals for PL‐NMA, though, were wider; this probably explains also their slightly better performance compared to the Wald‐type intervals in terms of coverage probability. As expected, the most uncertain results were obtained by the Bayesian random‐effects model which provided the widest credible intervals.

#### 3.4.5 Impact of all‐zero event studies

In 6 datasets, all other models but the PL‐NMA models provided implausible results with standard errors larger than $10^{8}$. Thus, all performance measures were calculated for all models after excluding those 6 scenarios.

For the PL‐NMA model, the inclusion or exclusion of all‐zero event studies do not seem to have important impact to the point estimates of the logORs, thus the results do not differ much in terms of bias. Bayesian models seem to have a poor performance, irrespective of the choice between inclusion or exclusion. In particular, we found that including all‐zero events in the Bayesian models can seriously bias the results. The decision between inclusion or exclusion of such studies appeared to have no impact for the BN‐NMA and common‐effect logistic regression model. Those models do not require such studies to be excluded prior to the analysis but they intrinsically exclude them.^8^

The PL‐NMA model and BN‐NMA estimated that $\hat{\varphi }=1\;$ and $\hat{\tau }=0\;$ across all the 1000 datasets of scenario 33 in both cases of inclusion or exclusion of all‐zero event studies. The Bayesian random‐effects model provided highly biased estimates of the heterogeneity parameter with mean bias equal to 0.26 and 0.27 when including and excluding all‐zero event studies, respectively.

Inclusion of all‐zero event studies seemed to slightly improve the performance of PL‐NMA model in terms of coverage probability. However, Wald‐type intervals tended to suffer from under‐coverage irrespectively of including or excluding while profile‐likelihood confidence intervals tended to provide coverage above the nominal level of 95%. Finally, the Bayesian models were suffering from under‐coverage; though their performance was improved when all‐zero event studies were included.

For the PL‐NMA model the MSE was slightly decreased when including all‐zero event studies while the Wald‐type confidence intervals were generally smaller. The mean length of profile likelihood confidence intervals remained robust and were not affected by the inclusion or the exclusion. For the Bayesian models the results did not differ when including or excluding all‐zero event studies. More details about the results of this scenario can be found in the Supplementary material S1.

## 4 CLINICAL EXAMPLES

### 4.1 Safety of inhaled medications for patients with chronic obstructive pulmonary disease

**FIGURE 5:**
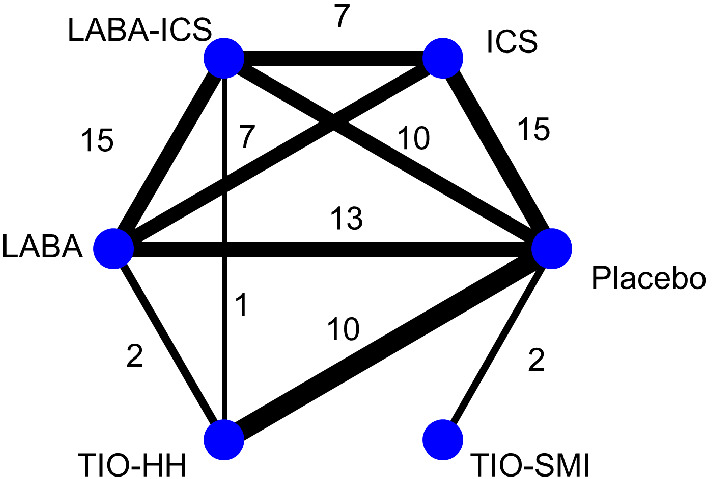
Network diagram for the inhaled medications example. HH, tiotropium dry powder; ICS, inhaled corticosteroid; LABA, long‐acting β2 agonist; TIO‐TIO‐SMI, tiotropium solution

We compared the results across the different models using a network that evaluates the safety of inhaled medications for patients with chronic obstructive pulmonary disease.^33^ The network consists of 41 studies and the outcome of interest is mortality. This is supposed to be a rare outcome as out of 52 462 patients only 2408 (4.6%) experienced the event of interest. In total, 13 out of 41 studies reported less than 5 events and 20 out of 99 arms had 0 events. The network diagram of this dataset is depicted in Figure 5. We analyzed the data using the PL‐NMA model both with Wald and profile likelihood CIs, the MH‐NMA model, the NCH‐NMA model and finally the same Bayesian models that were included in our simulation For the latter convergence was checked using the Brooks–Gelman–Rubin^32^ criterion. We did not use the IV model and the Bayesian random‐effects model with uniform heterogeneity prior due to the bad performance of these models in the simulation.

**FIGURE 6:**
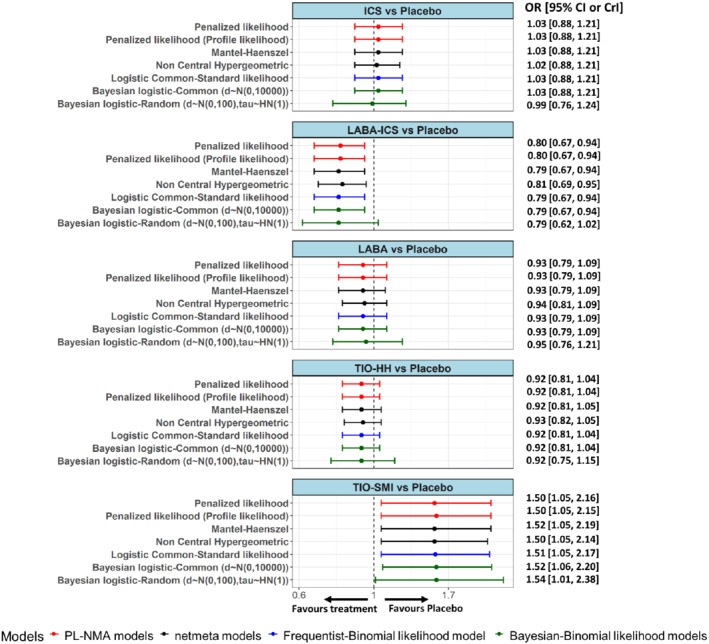
Forest plots showing the odds ratios obtained from the inhaled medications network for all comparisons against the reference

The results across the different NMA methods are almost identical (Figure 6). Regarding the estimation of heterogeneity, the additive parameter τwas estimated as zero for the BN‐NMA model and the multiplicative parameter φ was estimated as 1. However, the Bayesian random‐effects model resulted in $\hat{\tau }$= 0.12 [0, 0.35] for the model that assumes HN(1) prior.

### 4.2 Safety of different drug classes for chronic plaque psoriasis

**FIGURE 7:**
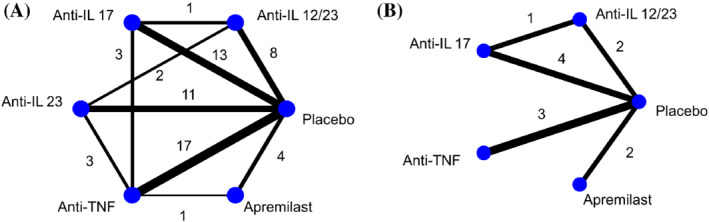
Network diagrams for the psoriasis example. Panel (A) shows the initially well‐connected network while panel (B) the resulting network after the exclusion of studies that report only zero events. The node Anti‐IL 23 is eliminated with the discarded studies. Anti‐IL, anti‐interleukin; Anti‐TNF, anti‐tumor necrosis factor

The second example is a network that evaluates the safety of different interventions for chronic plaque psoriasis.^34^ The dataset consists of 43 studies that involve 5 drug classes and placebo. Here, we compared again the different NMA methods but in a more extreme situation where the control group risks in the studies range between 0 and 1%. The outcome of interest is the number of malignancies that occurred after using the drugs. The mean sample size per study arm is 226 patients. Out of the 43 studies in this network, 15 (35%) are studies with zero events in all treatment groups. For MH‐NMA and NCH‐NMA the exclusion of all‐zero event arms leads to the removal of the treatment node Anti‐IL 23 from the network (Figure 7A,B). A more detailed explanation regarding the exclusion mechanism of those models can be found in our Supplementary material S1. The resulting network appears to be connected in terms of the rest treatments but also much reduced in total number of studies than the initial network.

**FIGURE 8:**
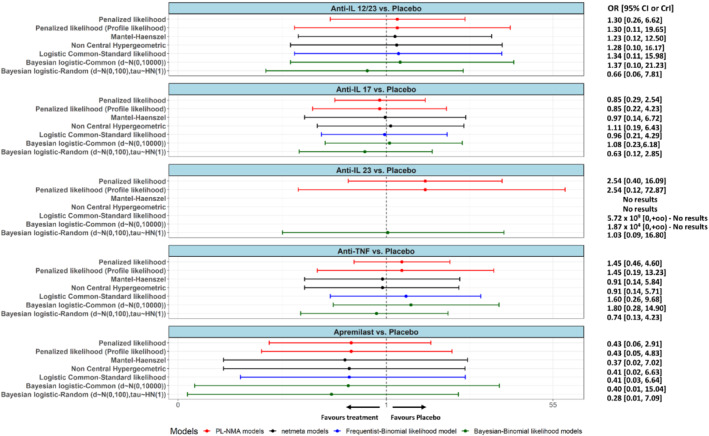
Forest plots showing the odds ratios obtained from psoriasis network for all comparisons against the reference

The results of the different approaches are shown in Figure 8. In terms of point estimates, for comparison Anti‐IL 12/23 vs Placebo all models except the Bayesian random‐effects models gave very similar results. For the comparison Apremilast vs Placebo, the results across all models appeared to be robust. For the rest of the comparisons there is a lot of diversity across the estimated point estimates which depicts the sensitivity of the results when the events are very rare. Finally, in all comparisons there are important differences across the Bayesian models which validates our hypothesis that the results are strongly dependent on the choice of prior distribution. With respect to heterogeneity, the PL‐NMA estimated the multiplicative term φ as 1. So, it suggests the absence of statistical heterogeneity. The Bayesian random‐effects model, though, gave $\hat{\tau }$= 0.56 (0.03, 1.61).

## 5 DISCUSSION

In this article, we have presented a new method for NMA of binary outcomes and rare events. Our method aims to improve the performance of existing NMA approaches for rare events in terms of bias and precision by using the penalized likelihood function for logistic regression that was originally proposed by Firth^13^ for individual studies. Our approach can only provide odds ratios. However, in the context of rare events, the differences between odds ratios and risk ratios are often negligible.^35^, ^36^

We evaluated the performance of the different methods and compared their results through an extended simulation study and two real clinical examples. We used various scenarios including studies with low or extremely low control group risks. The proposed PL‐NMA model appeared to have overall the best performance in terms of bias especially in situations with very few studies per comparison and very low control group risks. The performance on coverage probability was improved when profile‐likelihood confidence intervals were used but precision was reduced. In agreement with previous simulation studies,^4^ the IV‐NMA models are considered a suboptimal choice and we believe meta‐analysts should avoid their use for NMAs with rare events, especially for the cases with very small number of events per study (eg, <3). MH‐NMA, NCH‐NMA are good options under certain conditions, but they become less reliable as the network becomes sparser. BN‐NMA model is shown to be robust in terms of bias. However, we only evaluated the performance of the BN‐NMA model in scenarios with two‐arm studies as currently the model cannot take into account multivariate distributions for the random‐effects. The common‐effect Bayesian model performed well in terms of bias and coverage. The performance of the random‐effects Bayesian model was much improved across all the performance measures when we moved to narrower prior distributions for both the treatment effects and the heterogeneity parameters. In that case the model provided a reliable alternative as the results were less biased in comparison to the common‐effect Bayesian model and the coverage probability was usually above the nominal level. The estimates obtained from the penalized likelihood function are typically shrunk towards 0 and this in turn results in smaller standard errors. Hence, sometimes the PL‐NMA may underestimate the true study variance. As expected, all frequentist models failed to sufficiently frequently detect the presence of heterogeneity particularly for scenarios with extremely low control group risks (ie, 0.5%–1% and 1%–2%). On the other hand, Bayesian models identified some amount of heterogeneity but the estimates were in all cases highly biased. Of course the latter is a consequence coming from the choice of the prior distribution and other more suitable choices can potentially give less biased results.

An additional property of the proposed PL‐NMA model is the ability to synthesize all studies within a network of interventions irrespective of the number of events per arm since excluding such studies from the analysis may result in disconnected networks. The problem lies mainly in networks including studies with zero events in two or more treatment groups. To date, the optimal way to treat such studies in meta‐analysis or NMA is still unclear with some researchers arguing that they are informative^11^, ^37^ and others that those studies are non‐informative and problematic.^4^, ^36^ In scenario 33 of our simulation study, we intended to investigate the performance of the different models in the presence of several such studies. The choice of inclusion or exclusion appears to mostly affect the uncertainty measures such as the length of the confidence or credible intervals and the MSE which were typically smaller for all models when including all‐zero event studies. In terms of bias, the inclusion or exclusion appeared to have no serious impact for the PL‐NMA model. On the contrary, inclusion biased substantially the Bayesian models.

Although the PL‐NMA model is by nature a common‐effect model, we used a two‐stage approach for incorporating between‐study variance as a multiplicative overdispersion parameter. Multiplicative parameters are not the standard way to account for heterogeneity in meta‐analysis as typically an additive parameter is implemented. However, the penalized likelihood approach strongly relies on the analytical expression of the likelihood function and its moments; these are not available for the logistic regression model of Section 2.1 for the case of random‐effects. Thus, the incorporation of an additive heterogeneity parameter in the PL‐NMA method is not straightforward. The use of multiplicative heterogeneity parameters has been suggested previously for some meta‐analytical approaches^22^, ^23^ and particularly for meta‐analysis of rare events by Kuss^37^ and by Kulinskaya and Olkin.^38^ The latter, in contrast to our approach, is a one‐stage random‐effects model.^38^ A key difference between our two‐stage approach and the usual one‐stage random‐effects models is that the former does not affect the relative effect estimates but only inflates their variance when φ>1. In addition, the incorporation of the multiplicative parameter does not affect the weights of the studies and, thus, results might be dominated by the larger studies.^24^, ^39^ On the other hand, the additive heterogeneity model downweights larger studies. Hence, the additive model may be more plausible when larger studies have more heterogeneity while the multiplicative when the smaller studies do so.^40^, ^41^ A potential interpretation of the multiplicative model could be that the scale parameter represents a common variance and the study‐specific variances represent differing sample sizes. In our simulation, we found that all random‐effects approaches did not perform sufficiently well with respect to the estimation of the heterogeneity. It should be noted, though, that the Cochrane handbook suggests that estimation of heterogeneity is not of primary interest when performing meta‐analyzes of rare events and the priority should be the estimation of the treatment effects.^36^

Overall, the proposed PL‐NMA model offers a reliable choice for performing NMA for binary outcomes with rare events. Future work for NMA of rare events could consider the extension of the beta‐binomial model for meta‐analysis into NMA.^37^ Meta‐analysts should always bear in mind that the presence of studies with rare events makes estimation challenging and, therefore, a sensitivity analysis should be carried out to investigate the robustness of the results under various analysis schemes. It is planned to integrate the proposed PL‐NMA method into an R package.

## Supporting information


**Appendix S1** Supporting Information

## Data Availability

Data and R codes for the analysis of the two clinical examples can be found in the Supplementary material.
