# Supplementary material for: Network meta‐analysis of rare events using penalized likelihood regression
Source: Stat Med. 2022 Aug 26;41(26):5203–19. doi: 10.1002/sim.9562 (PMC9805041; doi:10.1002/sim.9562)
Supplement: Supplementary file 1 — Appendix S1 Supporting Information [file SIM-41-5203-s001.docx]

Supplementary material: Network meta-analysis of rare events using penalized likelihood regression

# Data generation mechanism for heterogeneous datasets

For generating heterogeneous datasets, we need to generate the study-specific true $logOR$s ($\delta_{i,1k}$) for the comparison of the $k\in K_{i}$ treatment versus the baseline treatment 1 in study $i$. To do so, we use the multivariate normal distribution, hence $\delta_{i,1k}\sim MVN(d_{1k},\boldsymbol{\Sigma}_{\mathbf{i}}$), $k\in K_{i}$. The matrix $\boldsymbol{\Sigma}_{\mathbf{i}}$ is the variance-covariance matrix of the random-effects with dimension $T_{i}\times T_{i}$, where $T_{i}$ represents the number of treatments in study $i$. The entries of the matrix $\boldsymbol{\Sigma}_{\mathbf{i}}$are assumed to be equal to $\tau^{2}$for its diagonal elements and $\frac{\tau^{2}}{2}$ for its off-diagonal elements. For example, for a 3-arm study after following the general procedure the true $logOR$s will have values equal to 0, 0.5 and 1. Then assuming than $\tau=0.1$, the $\delta_{i,1k}$will be drawn from the multivariate normal distribution with mean equal to ${(0, 0.5, 1)}^{'}$ and variance-covariance matrix $\boldsymbol{\Sigma}_{\mathbf{i}}$ equal to $\left( \begin{matrix} 0.01 & 0.005 & 0.005 \\ 0.005 & 0.01 & 0.005 \\ 0.005 & 0.005 & 0.01 \end{matrix} \right)$.

Afterwards we can easily generate the odds, event probabilities and finally the number of events per study arm following the procedure described in the main manuscript.

# Evaluated models

We evaluated the following models: the common and random-effect(s) inverse-variance (IV)^1,2^ model, the Mantel-Haenszel^3^ (MH) and non-central hypergeometric^4^ (NCH) common-effect models, our penalized likelihood NMA model (PL-NMA), the Binomial-Normal^5^ (BN-NMA) model and finally the two Bayesian common and random-effect(s) logistic regression models^6–8^. A detailed description of our PL-NMA model exists in the main manuscript. In the main manuscript a description exists as well for the BN-NMA model and the Bayesian models. Here, we provide a brief description for the rest of the models.

## Inverse-variance model

Let $\boldsymbol{y}$ denote the vector of the observed $logOR$s in the $N$studies that form a network with $T$ treatments. Then, this model can be summarized by the equation

$$\boldsymbol{y}=\mathbf{X}\boldsymbol{\delta}+\boldsymbol{\varepsilon}+\boldsymbol{\zeta}$$

where $\mathbf{X}$ is the design matrix with entries 0, 1, and -1 that depends on the structure of the network, $\boldsymbol{\delta}$ is the vector of the true $logOR$s representing the relative effects of all the treatments in the network versus the reference treatment, $\boldsymbol{\varepsilon}$ is the vector of the random errors, and $\boldsymbol{\zeta}$ is the vector of the random-effects. The random errors and the random-effects are assumed to follow multivariate normal distributions $\boldsymbol{\varepsilon\sim}MVN(\boldsymbol{0,}\mathbf{S}\boldsymbol{)}$ and $\boldsymbol{\zeta\sim}MVN(\boldsymbol{0,}\boldsymbol{\Sigma}\boldsymbol{)}$, where $\mathbf{S}$ is a block-diagonal matrix representing the within-study variance-covariance matrix and $\boldsymbol{\Sigma}$ is the variance-covariance matrix of the random-effects. Note that the within-study variances in $\mathbf{S}$ are treated as fixed and known. The NMA estimates and their variance are obtained by, $\hat{\boldsymbol{\delta}}={\mathbf{(}\mathbf{X}^{\mathbf{'}}\mathbf{WX)}}^{-1}\mathbf{X}^{'}\mathbf{W}\boldsymbol{y}$ and $V$($\hat{\boldsymbol{\delta}})= {\boldsymbol{(}\mathbf{X}^{\mathbf{'}}\mathbf{WX}\boldsymbol{)}}^{-1}$ respectively, where $\mathbf{W=}{\mathbf{(S+}\boldsymbol{\Sigma}\mathbf{)}}^{\mathbf{-1}}\boldsymbol{.}$ By setting the matrix $\boldsymbol{\Sigma}$ to be the zero matrix the above random-effects model becomes the common-effect IV model.

## Mantel-Haenszel model

The model can be described in 3 stages. At the first stage, all studies are grouped according to their treatment design. At the second stage, the following procedure takes place for each of the different treatment designs. Within the design $q$with $N_{q}$ studies and $T_{q}$ treatments, the model uses the generalized expressions for calculating the MH odds-ratios^9^ and provides the relative treatment effect of the treatment $B$ versus the design reference treatment $A$ in design $q$ ($\hat{\theta}_{q,AB})$. That is

$$\hat{\theta}_{q,AB}=\frac{L_{A+}-L_{B+}}{T_{q}}$$

where $L_{A+}=\sum_{J=1}^{Tq} L_{AJ}, L_{AB=}\ln\left( \frac{C_{\mathrm{AB}}}{C_{BA}} \right)$, $C_{AB}$=$\sum_{i=1}^{N_{q}} l_{ABi}$, and $l_{ABi}=\frac{r_{Ai}\left( n_{Bi}-r_{Bi} \right)}{n_{+i}}$, with $r_{Ai}$ being the number of events for treatment A in study $i$ and design $q$, $n_{Bi}$ the sample size of treatment $B$ in study $i$ and design $q$, and $n_{+i}$ the total sample size for study $i$of design $q$. For more information on how we can calculate the variance-covariance within each design ($\mathbf{V}_{\mathbf{q}})$ we refer to the original publication^3^. At the end of stage two, we have obtained the vector $\hat{\boldsymbol{\theta}}$ which contains the ${\hat{\boldsymbol{\theta}}}_{\boldsymbol{q}}$ for all designs$q$ and the variance-covariance matrix $\mathbf{V}$ which is a block-diagonal matrix with diagonal elements the different sub-matrices $\mathbf{V}_{\mathbf{q}}$ and off-diagonal elements being equal to zero. At the final stage, we calculate the final NMA estimates (${\hat{\boldsymbol{\delta}}}_{MH}\mathbf{)}$ by synthesizing the MH odds-ratios across all the designs using, that ${\hat{\boldsymbol{\delta}}}_{MH}={\mathbf{(}\mathbf{X}^{\mathbf{'}}\mathbf{VX)}}^{-1}\mathbf{X}^{'}\mathbf{V}\boldsymbol{\theta}$ with variance-covariance matrix $\mathbf{V}\left( {\hat{\boldsymbol{\delta}}}_{MH} \right)={\boldsymbol{(}\mathbf{X}^{\mathbf{'}}\mathbf{VX)}}^{-1}$.

## Non-central hypergeometric model

This model is based on the within-study distributional assumption of non-central hypergeometric distribution. Specifically, given the total number of events $R_{i}$in a study i, i.e. $R_{i}=\sum_{k\in K_{i}} r_{ik}$, the vector $\boldsymbol{r=}\left[ r_{ik} \right], k\in K_{i}-\{1\}$ follows a multivariate non-central hypergeometric distribution. For a 3-arm study the likelihood function can be written as

$$\frac{exp(r_{i2}\delta_{12}+r_{i3}\delta_{13})}{\sum_{j^{(2)},j^{(3)}} \left( \begin{matrix} n_{i2} \\ j^{(2)} \end{matrix} \right)\left( \begin{matrix} n_{i3} \\ j^{(3)} \end{matrix} \right)\left( \begin{matrix} n_{i1} \\ R_{i}-j^{(2)}-j^{(3)} \end{matrix} \right)exp(j^{\left( 2 \right)}\delta_{12}+j^{\left( 3 \right)}\delta_{13})}$$

where the sum in the denominator is over all pairs ($j^{(2)},j^{(3)})$ for which the binomial coefficients can be defined. The version implemented in netmeta^10^ and used in our simulations is based on the Breslow’s approximation to the above likelihood which is valid when the outcome of interest is rare. Using this approximation, the likelihood function becomes

$$\frac{exp(r_{i2}\delta_{12}+r_{i3}\delta_{13})}{\left[ (n_{i1}+n_{i2}\exp\left( \delta_{12} \right)+n_{i3}\exp\left( \delta_{13} \right) \right]^{R_{i}}}$$

| **#** | **IV-Common** | | | **IV-Random** | | | **MH** | | | **NCH** | | | **PL-NMA-Common** | | | **PL-NMA- Random** | | | **Binomial Normal** | | | **Logistic NMA (Standard likelihood-Common)** | | |
| --- | --- | --- | --- | --- | --- | --- | --- | --- | --- | --- | --- | --- | --- | --- | --- | --- | --- | --- | --- | --- | --- | --- | --- | --- |
|  | Mean bias | MSE | Coverage  (%) | Mean bias | MSE | Coverage  (%) | Mean bias | MSE | Coverage  (%) | Mean bias | MSE | Coverage  (%) | Mean bias | MSE | Coverage  (Wald-**Prof. Lik, %**) | Mean bias | MSE | Coverage  (%) | Mean bias | MSE | Coverage  (%) | Mean bias | MSE | Coverage  (%) |
| 1 | -0.07 | 0.34 | 97.80 | -0.07 | 0.35 | 98.00 | -0.04 | 0.37 | 96.90 | -0.04 | 0.34 | 96.20 | 0.01 | 0.35 | 95.00  **95.30** | 0.01 | 0.35 | 95.20 | 0.04 | 0.38 | 95.00 | 0.04 | 0.38 | 95.00 |
| 2 | -0.07 | 0.35 | 97.30 | -0.07 | 0.36 | 97.30 | -0.05 | 0.37 | 96.40 | -0.05 | 0.35 | 95.60 | 0.00 | 0.36 | 94.00  **94.50** | 0.00 | 0.36 | 94.00 | 0.03 | 0.40 | 94.40 | 0.03 | 0.40 | 94.40 |
| 3 | -0.03 | 0.20 | 97.00 | -0.03 | 0.21 | 97.30 | 0.00 | 0.20 | 95.80 | -0.05 | 0.18 | 95.40 | 0.01 | 0.20 | 94.60  **94.70** | 0.01 | 0.20 | 95.00 | 0.03 | 0.21 | 94.50 | 0.03 | 0.21 | 94.50 |
| 4 | -0.04 | 0.21 | 96.40 | -0.04 | 0.22 | 96.60 | -0.01 | 0.21 | 95.40 | -0.06 | 0.18 | 94.60 | 0.00 | 0.20 | 94.20  **94.30** | 0.00 | 0.21 | 94.60 | 0.02 | 0.21 | 93.80 | 0.02 | 0.21 | 93.80 |
| 5 | -0.09 | 0.17 | 97.40 | -0.08 | 0.17 | 97.40 | -0.01 | 0.17 | 95.70 | -0.03 | 0.16 | 95.40 | 0.00 | 0.17 | 94.50  **95.00** | 0.00 | 0.17 | 94.50 | 0.02 | 0.18 | 94.90 | 0.02 | 0.18 | 94.90 |
| 6 | -0.07 | 0.17 | 97.30 | -0.07 | 0.18 | 97.40 | 0.00 | 0.18 | 95.60 | -0.02 | 0.17 | 95.10 | 0.01 | 0.17 | 94.20  **94.80** | 0.01 | 0.17 | 94.20 | 0.03 | 0.19 | 94.50 | 0.03 | 0.19 | 94.50 |
| 7 | -0.05 | 0.10 | 96.50 | -0.05 | 0.10 | 96.80 | 0.00 | 0.10 | 95.40 | -0.06 | 0.09 | 94.40 | 0.00 | 0.10 | 94.80  **95.20** | 0.00 | 0.10 | 95.00 | 0.01 | 0.10 | 94.70 | 0.01 | 0.10 | 94.70 |
| 8 | -0.04 | 0.10 | 96.20 | -0.04 | 0.10 | 96.50 | 0.00 | 0.10 | 95.00 | -0.06 | 0.09 | 94.30 | 0.00 | 0.10 | 94.30  **94.60** | 0.00 | 0.10 | 94.30 | 0.02 | 0.10 | 94.30 | 0.02 | 0.10 | 94.30 |
| 9 | -0.08 | 0.22 | 97.80 | -0.08 | 0.22 | 97.80 | -0.06 | 0.22 | 96.90 | -0.06 | 0.21 | 96.00 | -0.01 | 0.21 | 94.80  **95.50** | -0.01 | 0.21 | 94.80 | 0.01 | 0.23 | 95.30 | 0.01 | 0.23 | 95.30 |
| 10 | -0.07 | 0.21 | 98.00 | -0.07 | 0.22 | 98.10 | -0.04 | 0.22 | 97.20 | -0.04 | 0.20 | 96.00 | 0.01 | 0.21 | 94.50  **95.30** | 0.01 | 0.21 | 94.50 | 0.03 | 0.23 | 95.00 | 0.03 | 0.23 | 95.00 |
| 11 | -0.07 | 0.27 | 97.60 | -0.06 | 0.27 | 97.70 | -0.03 | 0.28 | 96.70 | -0.01 | 0.27 | 95.40 | 0.00 | 0.26 | 94.70  **95.00** | 0.00 | 0.27 | 94.80 | 0.02 | 0.29 | 94.90 | 0.02 | 0.29 | 94.90 |
| 12 | -0.06 | 0.27 | 97.10 | -0.06 | 0.28 | 97.20 | -0.03 | 0.28 | 96.30 | -0.01 | 0.27 | 95.20 | 0.00 | 0.27 | 94.10  **94.60** | 0.00 | 0.27 | 94.40 | 0.02 | 0.29 | 94.60 | 0.02 | 0.29 | 94.60 |
| 13 | -0.12 | 0.48 | 98.30 | -0.12 | 0.48 | 98.30 | -0.12 | 0.61 | 97.70 | -0.10 | 0.59 | 97.00 | 0.00 | 0.52 | 94.50  **95.00** | 0.00 | 0.52 | 94.50 | 0.04 | 0.62 | 95.20 | 0.04 | 0.62 | 95.20 |
| 14 | -0.12 | 0.48 | 98.20 | -0.12 | 0.48 | 98.20 | -0.12 | 0.61 | 97.40 | -0.09 | 0.60 | 96.40 | 0.00 | 0.53 | 94.00  **94.80** | 0.00 | 0.53 | 94.00 | 0.04 | 0.63 | 94.90 | 0.04 | 0.63 | 94.90 |
| 15 | -0.14 | 0.25 | 97.90 | -0.14 | 0.25 | 97.90 | -0.05 | 0.27 | 96.50 | -0.02 | 0.27 | 95.40 | -0.02 | 0.24 | 94.00  **95.20** | -0.02 | 0.24 | 94.00 | 0.00 | 0.29 | 95.10 | 0.00 | 0.29 | 95.10 |
| 16 | -0.12 | 0.24 | 97.80 | -0.12 | 0.24 | 97.80 | -0.02 | 0.27 | 96.60 | 0.01 | 0.27 | 95.10 | 0.01 | 0.25 | 93.30  **94.50** | 0.01 | 0.25 | 93.30 | 0.03 | 0.28 | 94.40 | 0.03 | 0.28 | 94.40 |
| 17 | -0.07 | 0.17 | 96.40 | -0.07 | 0.18 | 96.80 | 0.04 | 0.19 | 95.60 | 0.01 | 0.18 | 95.60 | 0.02 | 0.18 | 95.40  **95.60** | 0.02 | 0.18 | 95.60 | **-** | **-** | **-** | 0.03 | 0.19 | 95.60 |
| 18 | -0.09 | 0.17 | 96.20 | -0.09 | 0.17 | 96.60 | 0.01 | 0.18 | 95.40 | -0.01 | 0.17 | 95.60 | -0.01 | 0.17 | 95.20  **95.20** | -0.01 | 0.17 | 95.30 | **-** | **-** | **-** | 0.01 | 0.18 | 95.40 |
| 19 | -0.14 | 0.30 | 97.20 | -0.14 | 0.30 | 97.20 | 0.05 | 0.40 | 96.40 | 0.05 | 0.39 | 96.40 | 0.02 | 0.34 | 96.20  **95.80** | 0.02 | 0.34 | 96.20 | **-** | **-** | **-** | 0.06 | 0.40 | 96.30 |
| 20 | -0.15 | 0.30 | 97.50 | -0.15 | 0.30 | 97.60 | 0.03 | 0.37 | 96.00 | 0.02 | 0.37 | 96.00 | -0.01 | 0.33 | 95.60  **95.50** | -0.01 | 0.33 | 95.60 | **-** | **-** | **-** | 0.03 | 0.37 | 96.00 |
| 21 | -0.04 | 0.10 | 96.20 | -0.03 | 0.10 | 96.60 | 0.02 | 0.10 | 95.80 | -0.02 | 0.10 | 95.60 | 0.01 | 0.10 | 95.80  **96.00** | 0.01 | 0.10 | 96.20 | **-** | **-** | **-** | 0.02 | 0.10 | 95.80 |
| 22 | -0.05 | 0.10 | 96.10 | -0.05 | 0.10 | 96.60 | 0.00 | 0.10 | 95.50 | -0.04 | 0.09 | 95.40 | 0.00 | 0.10 | 95.40  **95.60** | 0.00 | 0.10 | 95.70 | **-** | **-** | **-** | 0.00 | 0.10 | 95.40 |
| 23 | -0.01 | 0.06 | 95.30 | -0.01 | 0.06 | 96.30 | 0.01 | 0.06 | 94.80 | -0.06 | 0.05 | 92.10 | 0.01 | 0.06 | 94.80  **94.90** | 0.01 | 0.06 | 95.50 | **-** | **-** | **-** | 0.02 | 0.06 | 94.80 |
| 24 | -0.02 | 0.05 | 95.70 | -0.02 | 0.06 | 96.60 | 0.00 | 0.05 | 95.30 | -0.07 | 0.05 | 92.40 | 0.00 | 0.05 | 95.20  **95.40** | 0.00 | 0.06 | 95.50 | **-** | **-** | **-** | 0.00 | 0.05 | 95.30 |
| 25 | -0.07 | 0.18 | 95.60 | -0.07 | 0.18 | 95.90 | 0.02 | 0.19 | 95.20 | 0.00 | 0.19 | 95.40 | 0.00 | 0.18 | 95.30  **95.20** | 0.00 | 0.18 | 95.30 | **-** | **-** | **-** | 0.02 | 0.19 | 95.20 |
| 26 | -0.06 | 0.17 | 96.70 | -0.05 | 0.18 | 96.90 | 0.03 | 0.19 | 95.70 | 0.02 | 0.18 | 95.90 | 0.02 | 0.18 | 95.70  **95.40** | 0.02 | 0.18 | 95.80 | **-** | **-** | **-** | 0.03 | 0.19 | 95.60 |
| 27 | -0.14 | 0.30 | 96.20 | -0.14 | 0.30 | 96.20 | 0.04 | 0.42 | 95.40 | 0.03 | 0.42 | 95.50 | 0.01 | 0.37 | 95.30  **94.80** | 0.01 | 0.37 | 95.30 | **-** | **-** | **-** | 0.04 | 0.42 | 95.40 |
| 28 | -0.13 | 0.29 | 96.70 | -0.13 | 0.29 | 96.80 | 0.05 | 0.40 | 96.40 | 0.04 | 0.40 | 96.40 | 0.02 | 0.36 | 96.30  **95.60** | 0.02 | 0.36 | 96.30 | **-** | **-** | **-** | 0.05 | 0.40 | 96.30 |
| 29 | -0.04 | 0.10 | 95.60 | -0.04 | 0.10 | 96.10 | 0.01 | 0.11 | 95.20 | -0.02 | 0.10 | 95.40 | 0.00 | 0.10 | 95.20  **95.20** | 0.00 | 0.10 | 95.30 | **-** | **-** | **-** | 0.01 | 0.11 | 95.20 |
| 30 | -0.03 | 0.10 | 96.00 | -0.03 | 0.10 | 96.70 | 0.02 | 0.11 | 95.60 | -0.01 | 0.10 | 95.50 | 0.01 | 0.10 | 95.60  **95.60** | 0.01 | 0.10 | 95.90 | **-** | **-** | **-** | 0.02 | 0.11 | 95.60 |
| 31 | -0.02 | 0.06 | 95.60 | -0.02 | 0.06 | 96.00 | 0.00 | 0.06 | 94.70 | -0.06 | 0.06 | 93.10 | 0.00 | 0.06 | 94.90  **94.80** | 0.00 | 0.06 | 95.30 | **-** | **-** | **-** | 0.00 | 0.06 | 94.80 |
| 32 | -0.01 | 0.06 | 95.50 | -0.01 | 0.06 | 96.10 | 0.01 | 0.06 | 94.70 | -0.05 | 0.06 | 93.90 | 0.01 | 0.06 | 94.70  **94.60** | 0.01 | 0.06 | 95.30 | **-** | **-** | **-** | 0.01 | 0.06 | 94.60 |

**S-Table 1:** Results in terms of mean bias, mean square error (MSE) and coverage probability (%) across all 32 scenarios and across all frequentist models

**S-Table 2:** Results in terms of mean bias, mean square error (MSE) and coverage probability (%) across all 32 scenarios and across Bayesian models.

| **#** | **Bayesian** $\boldsymbol{(d\sim N}\left( \boldsymbol{0,10}\boldsymbol{0}^{\boldsymbol{2}} \right)\boldsymbol{)}$ | | | **Bayesian** $\boldsymbol{(d\sim N}\left( \boldsymbol{0,10}\boldsymbol{0}^{\boldsymbol{2}} \right)\boldsymbol{,}\boldsymbol{\tau}\boldsymbol{\sim}\boldsymbol{U}\left( \boldsymbol{0}\boldsymbol{,}\boldsymbol{2} \right)\boldsymbol{)}$ | | | **Bayesian** $\boldsymbol{(d\sim N}\left( \boldsymbol{0,1}\boldsymbol{0}^{\boldsymbol{2}} \right)\boldsymbol{,}\boldsymbol{\tau}\boldsymbol{\sim}\boldsymbol{HN}\left( \boldsymbol{1} \right)\boldsymbol{)}$ | | |
| --- | --- | --- | --- | --- | --- | --- | --- | --- | --- |
|  | **Mean bias** | **MSE** | **Coverage**  **(%)** | **Mean bias** | **MSE** | **Coverage**  **(%)** | **Mean bias** | **MSE** | **Coverage (%)** |
| 1 | 0.07 | 0.42 | 93.60 | 0.11 | 0.58 | 96.50 | 0.04 | 0.43 | 95.20 |
| 2 | 0.06 | 0.43 | 93.10 | 0.10 | 0.61 | 96.10 | 0.04 | 0.44 | 94.70 |
| 3 | 0.04 | 0.22 | 93.70 | 0.06 | 0.28 | 96.70 | 0.04 | 0.24 | 95.40 |
| 4 | 0.04 | 0.23 | 93.30 | 0.05 | 0.29 | 96.50 | 0.03 | 0.24 | 95.10 |
| 5 | 0.03 | 0.19 | 94.10 | 0.05 | 0.23 | 96.00 | 0.02 | 0.20 | 95.30 |
| 6 | 0.05 | 0.20 | 93.70 | 0.06 | 0.24 | 95.90 | 0.03 | 0.20 | 95.00 |
| 7 | 0.02 | 0.10 | 94.50 | 0.03 | 0.12 | 96.40 | 0.02 | 0.11 | 95.70 |
| 8 | 0.03 | 0.11 | 93.50 | 0.04 | 0.12 | 95.70 | 0.02 | 0.12 | 95.20 |
| 9 | 0.03 | 0.24 | 94.60 | 0.04 | 0.28 | 96.10 | 0.01 | 0.25 | 95.50 |
| 10 | 0.05 | 0.24 | 94.40 | 0.06 | 0.28 | 96.00 | 0.03 | 0.25 | 95.50 |
| 11 | 0.04 | 0.30 | 93.60 | 0.07 | 0.41 | 96.80 | 0.02 | 0.32 | 95.50 |
| 12 | 0.05 | 0.31 | 93.60 | 0.08 | 0.43 | 95.50 | 0.03 | 0.33 | 95.40 |
| 13 | 0.09 | 0.70 | 93.20 | 0.17 | 1.08 | 95.60 | 0.02 | 0.67 | 94.60 |
| 14 | 0.09 | 0.72 | 92.90 | 0.16 | 1.11 | 96.00 | 0.02 | 0.68 | 94.60 |
| 15 | 0.02 | 0.29 | 94.40 | 0.05 | 0.38 | 96.30 | -0.03 | 0.29 | 95.20 |
| 16 | 0.05 | 0.30 | 93.70 | 0.09 | 0.40 | 95.80 | 0.00 | 0.30 | 95.00 |
| 17 | 0.05 | 0.20 | 94.70 | 0.07 | 0.26 | 97.30 | 0.03 | 0.20 | 96.40 |
| 18 | 0.02 | 0.18 | 95.20 | 0.05 | 0.24 | 97.30 | 0.01 | 0.19 | 96.40 |
| 19 | 0.10 | 0.45 | 94.30 | 0.15 | 0.61 | 97.00 | 0.03 | 0.41 | 96.00 |
| 20 | 0.06 | 0.41 | 94.90 | 0.11 | 0.57 | 97.00 | 0.01 | 0.39 | 95.90 |
| 21 | 0.03 | 0.11 | 95.70 | 0.04 | 0.13 | 97.80 | 0.02 | 0.11 | 97.20 |
| 22 | 0.01 | 0.10 | 95.00 | 0.02 | 0.13 | 97.70 | 0.01 | 0.11 | 97.20 |
| 23 | 0.02 | 0.06 | 94.70 | 0.03 | 0.07 | 97.60 | 0.02 | 0.07 | 96.70 |
| 24 | 0.01 | 0.06 | 95.20 | 0.01 | 0.07 | 97.70 | 0.01 | 0.06 | 97.00 |
| 25 | 0.03 | 0.21 | 94.50 | 0.03 | 0.23 | 96.20 | 0.00 | 0.20 | 95.60 |
| 26 | 0.05 | 0.20 | 95.00 | 0.05 | 0.23 | 96.50 | 0.02 | 0.20 | 95.80 |
| 27 | 0.07 | 0.48 | 93.50 | 0.08 | 0.55 | 95.60 | 0.00 | 0.43 | 94.90 |
| 28 | 0.09 | 0.45 | 94.50 | 0.10 | 0.52 | 96.30 | 0.01 | 0.41 | 95.80 |
| 29 | 0.02 | 0.11 | 94.80 | 0.02 | 0.12 | 96.50 | 0.00 | 0.11 | 96.10 |
| 30 | 0.03 | 0.11 | 94.80 | 0.03 | 0.12 | 96.90 | 0.02 | 0.12 | 96.70 |
| 31 | 0.01 | 0.06 | 94.50 | 0.01 | 0.07 | 96.50 | 0.00 | 0.06 | 96.30 |
| 32 | 0.01 | 0.06 | 94.20 | 0.01 | 0.07 | 96.40 | 0.01 | 0.07 | 96.40 |

**S-Table 3:** Extent of studies with zero events in all treatment arms in the simulation study.

| Scenario | Minimum | 1st Quartile | Median | 3rd Quartile | Maximum | Total number of studies per dataset |
| --- | --- | --- | --- | --- | --- | --- |
| 1 | 0 | 0 | 0 | 0 | 3 | 20 |
| 2 | 0 | 0 | 0 | 0 | 2 | 20 |
| 3 | 0 | 0 | 0 | 0 | 1 | 20 |
| 4 | 0 | 0 | 0 | 0 | 1 | 20 |
| 5 | 0 | 0 | 0 | 0 | 3 | 40 |
| 6 | 0 | 0 | 0 | 1 | 4 | 40 |
| 7 | 0 | 0 | 0 | 0 | 1 | 40 |
| 8 | 0 | 0 | 0 | 0 | 1 | 40 |
| 9 | 0 | 0 | 0 | 1 | 3 | 56 |
| 10 | 0 | 0 | 0 | 1 | 3 | 56 |
| 11 | 0 | 0 | 0 | 0 | 2 | 20 |
| 12 | 0 | 0 | 0 | 0 | 1 | 20 |
| 13 | 0 | 0 | 1 | 1 | 4 | 20 |
| 14 | 0 | 0 | 1 | 1 | 5 | 20 |
| 15 | 0 | 1 | 1 | 2 | 6 | 40 |
| 16 | 0 | 1 | 1 | 2 | 6 | 40 |
| 17 | 0 | 0 | 0 | 0 | 1 | 8 |
| 18 | 0 | 0 | 0 | 0 | 0 | 8 |
| 19 | 0 | 0 | 0 | 0 | 1 | 8 |
| 20 | 0 | 0 | 0 | 0 | 2 | 8 |
| 21 | 0 | 0 | 0 | 0 | 1 | 8 |
| 22 | 0 | 0 | 0 | 0 | 1 | 8 |
| 23 | 0 | 0 | 0 | 0 | 1 | 8 |
| 24 | 0 | 0 | 0 | 0 | 1 | 8 |
| 25 | 0 | 0 | 0 | 0 | 0 | 8 |
| 26 | 0 | 0 | 0 | 0 | 0 | 8 |
| 27 | 0 | 0 | 0 | 0 | 2 | 8 |
| 28 | 0 | 0 | 0 | 0 | 1 | 8 |
| 29 | 0 | 0 | 0 | 0 | 1 | 8 |
| 30 | 0 | 0 | 0 | 0 | 1 | 8 |
| 31 | 0 | 0 | 0 | 0 | 0 | 8 |
| 32 | 0 | 0 | 0 | 0 | 1 | 8 |
| 33 | 6 | 13 | 15 | 17 | 25 | 40 |

### **
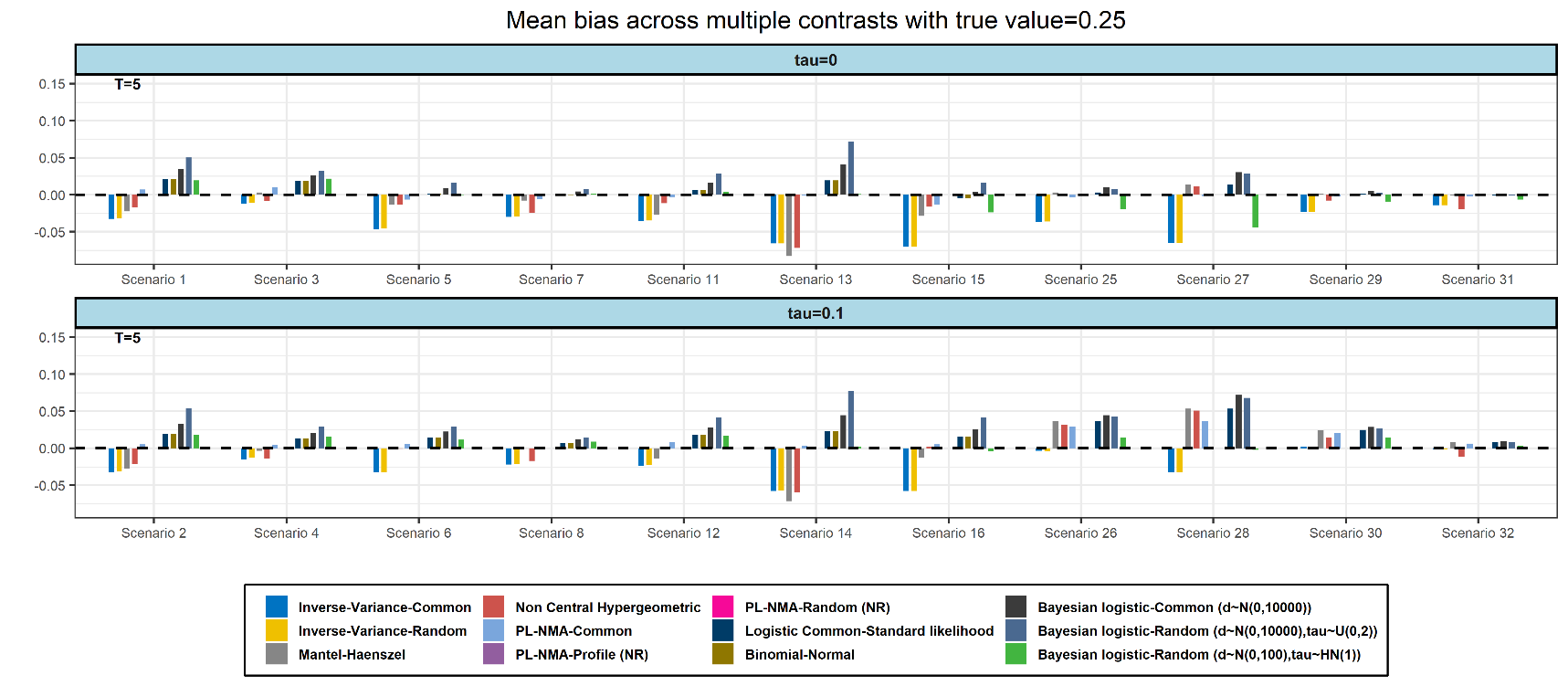
**Results in terms of true $logORs$

**S-Figure 1**: Results in terms of bias when true $logOR$ is equal to 0.25. Models marked as NR are not relevant to the figure and thus no results are plotted.

**
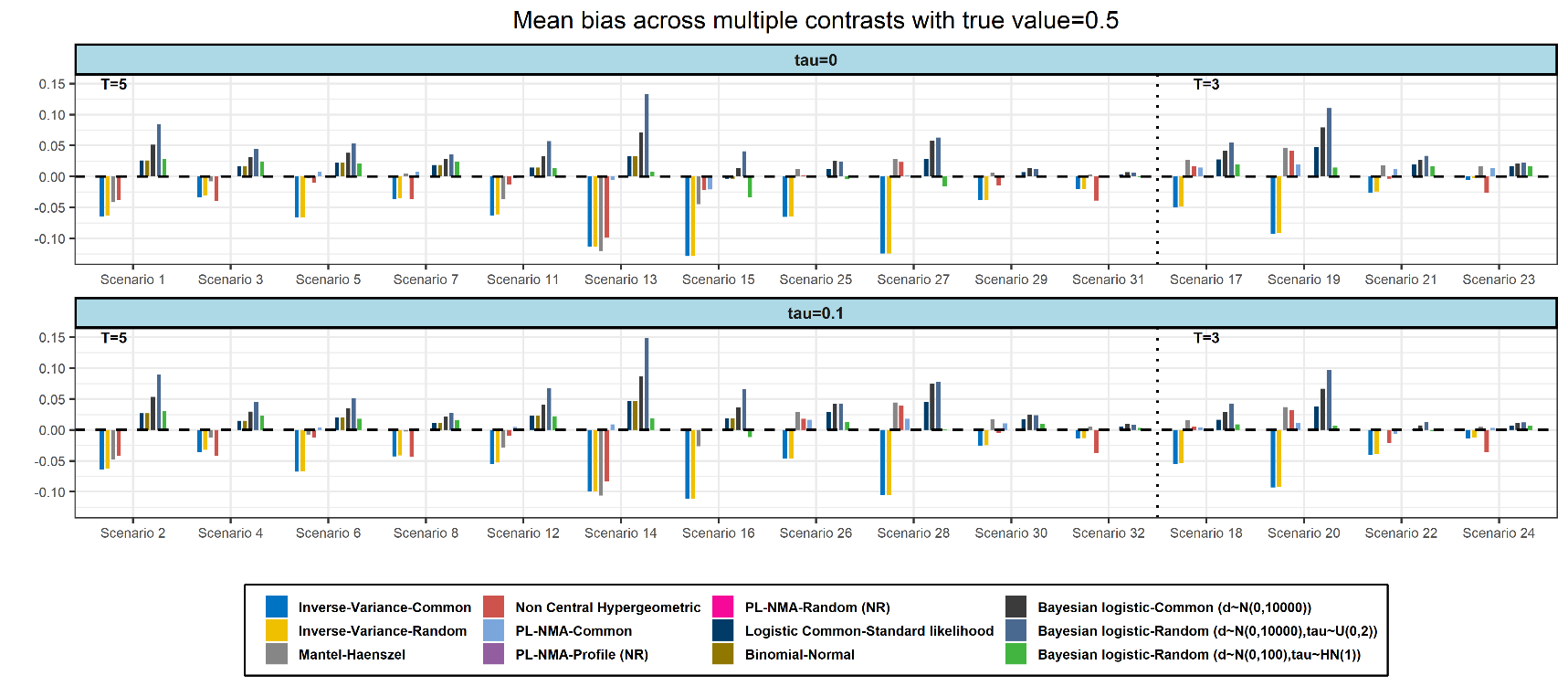
**

**S-Figure 2**: Results in terms of bias when true $logOR$ is equal to 0.50. Models marked as NR are not relevant to the figure and thus no results are plotted.

**
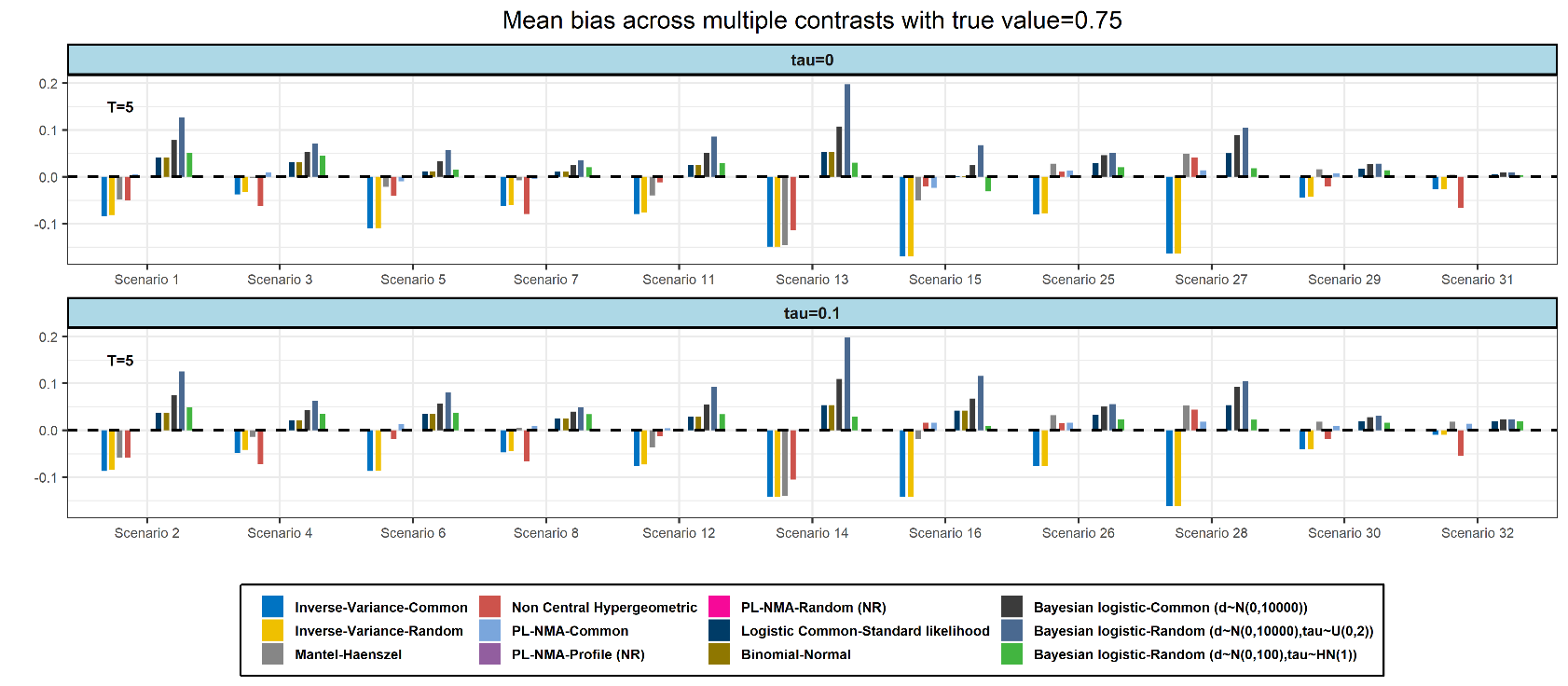
**

**
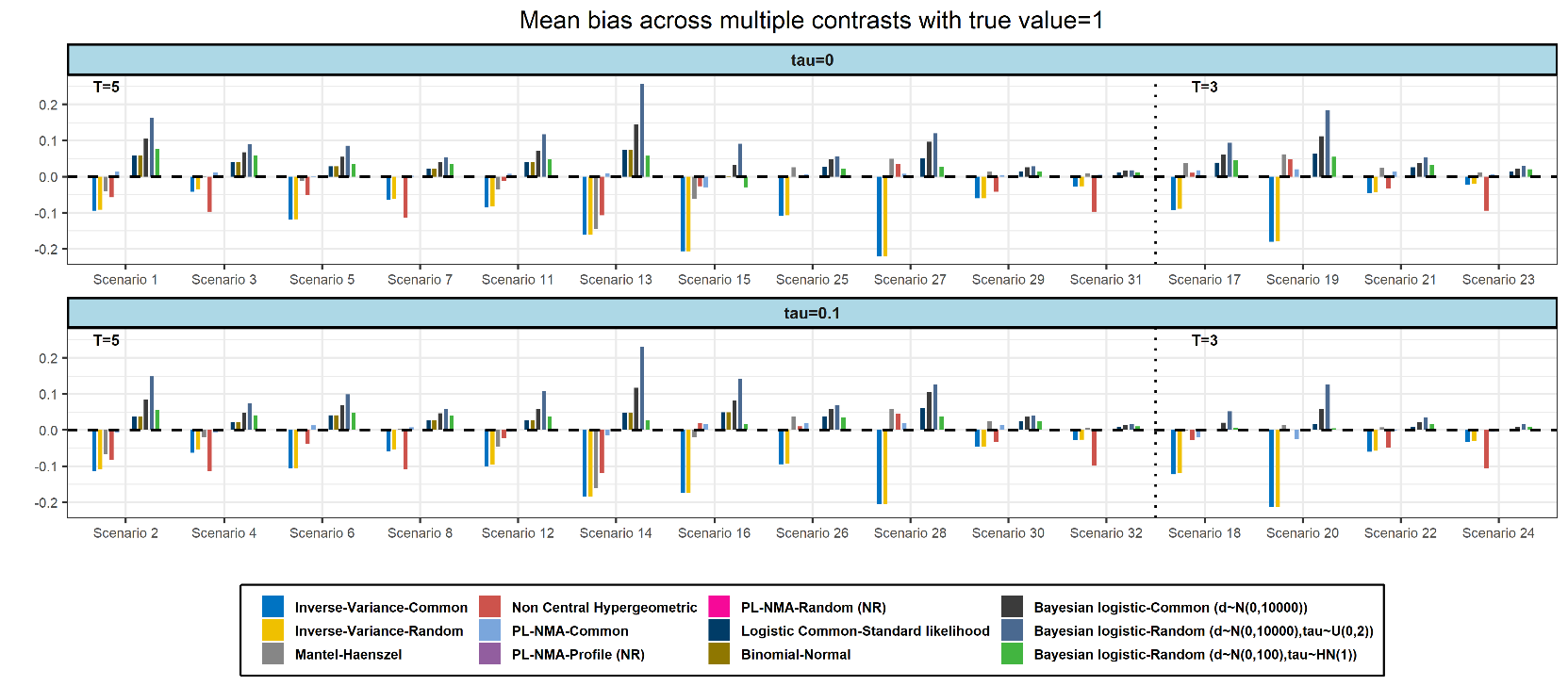
**

**S-Figure 3:** Results in terms of bias when true $logOR$ is equal to 0.75. Models marked as NR are not relevant to the figure and thus no results are plotted.

**S-Figure 4**: Results in terms of bias when true $logOR$ is equal to 1. Models marked as NR are not relevant to the figure and thus no results are plotted.

### Results for Scenario 33


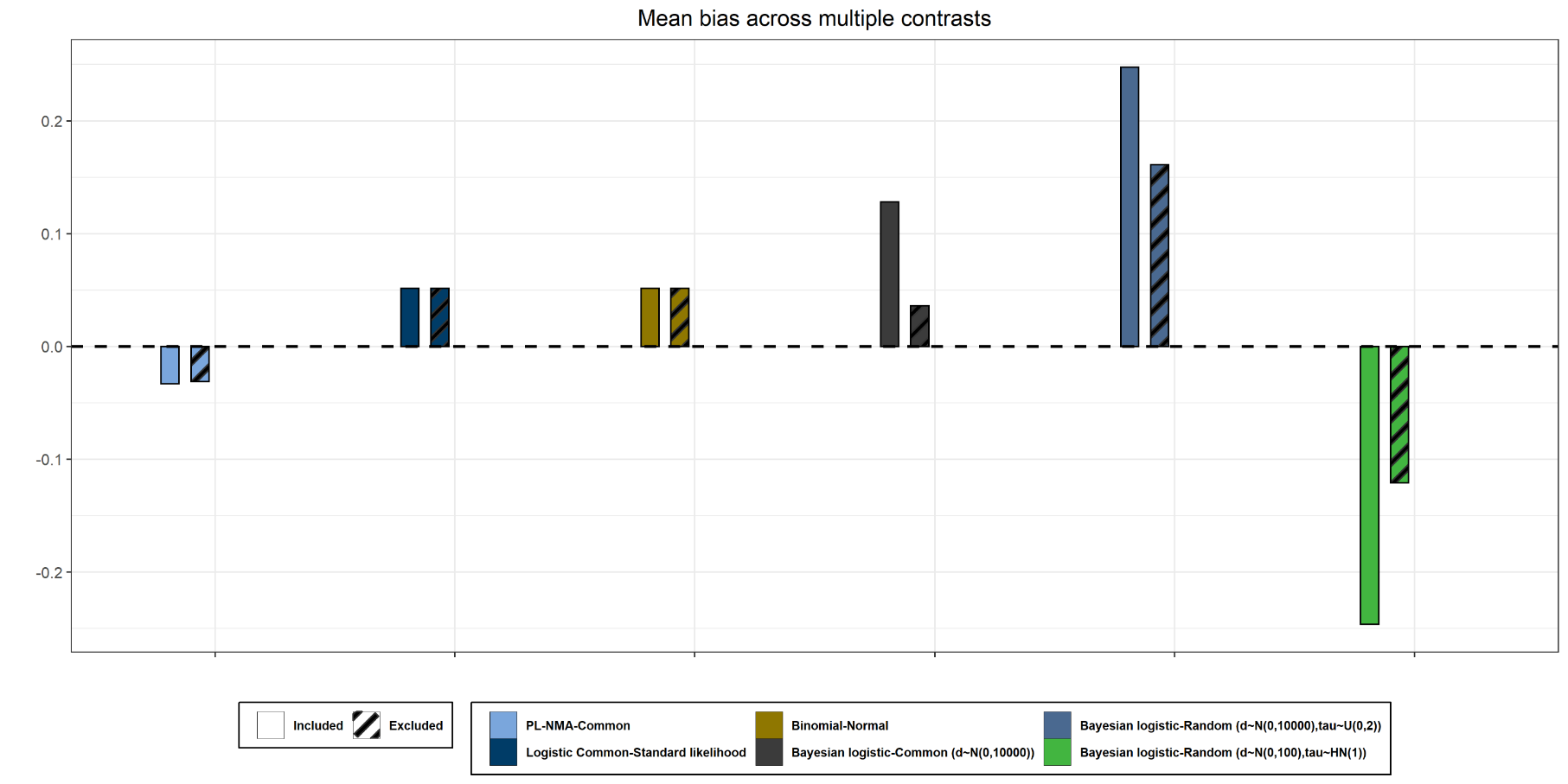


**S-Figure 5:** Mean bias across multiple contrasts for Scenario 33.


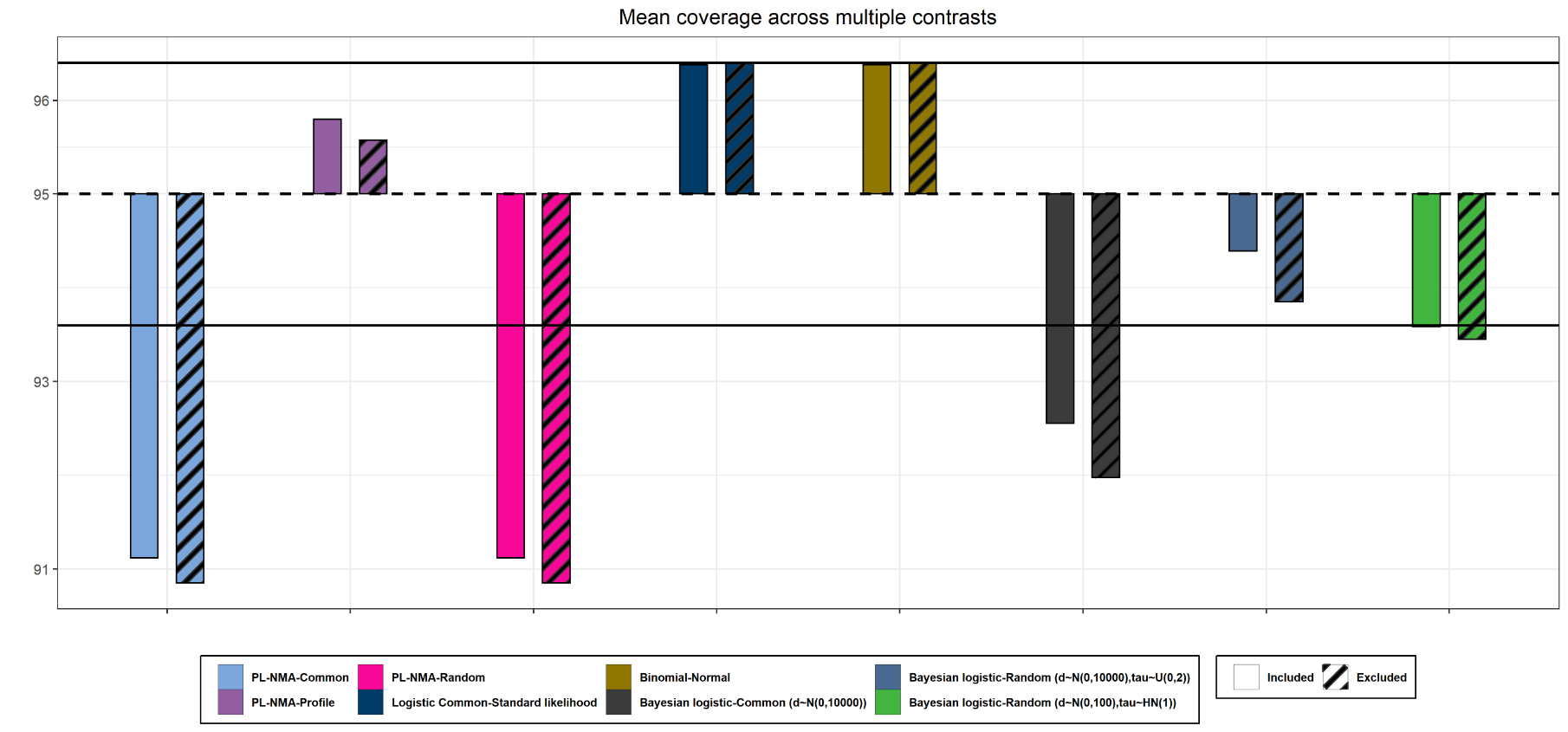


**S-Figure 6:** Mean coverage across multiple contrasts for Scenario 33.


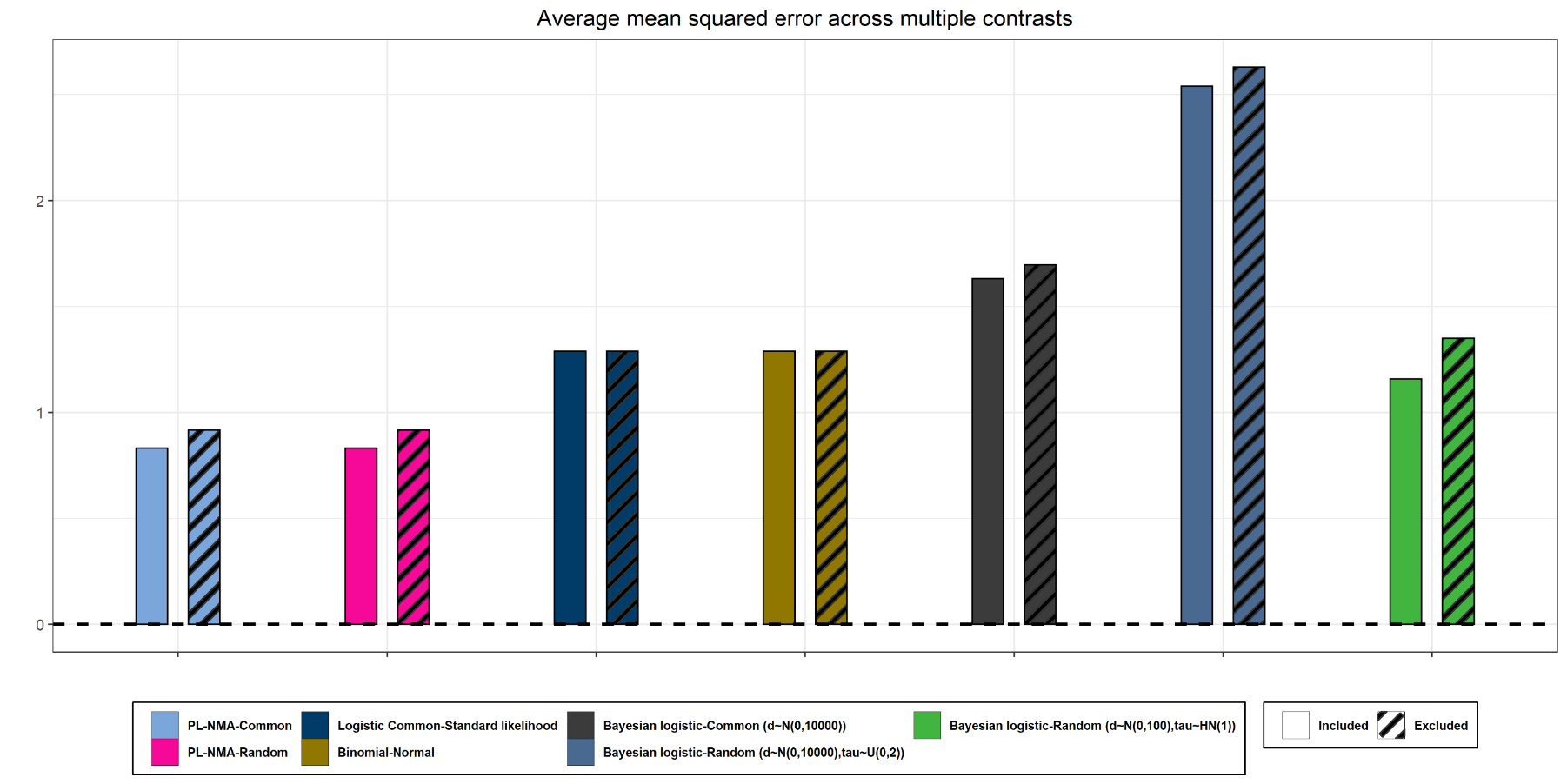


**S-Figure 7:** Average mean squared error across multiple contrasts for Scenario 33.


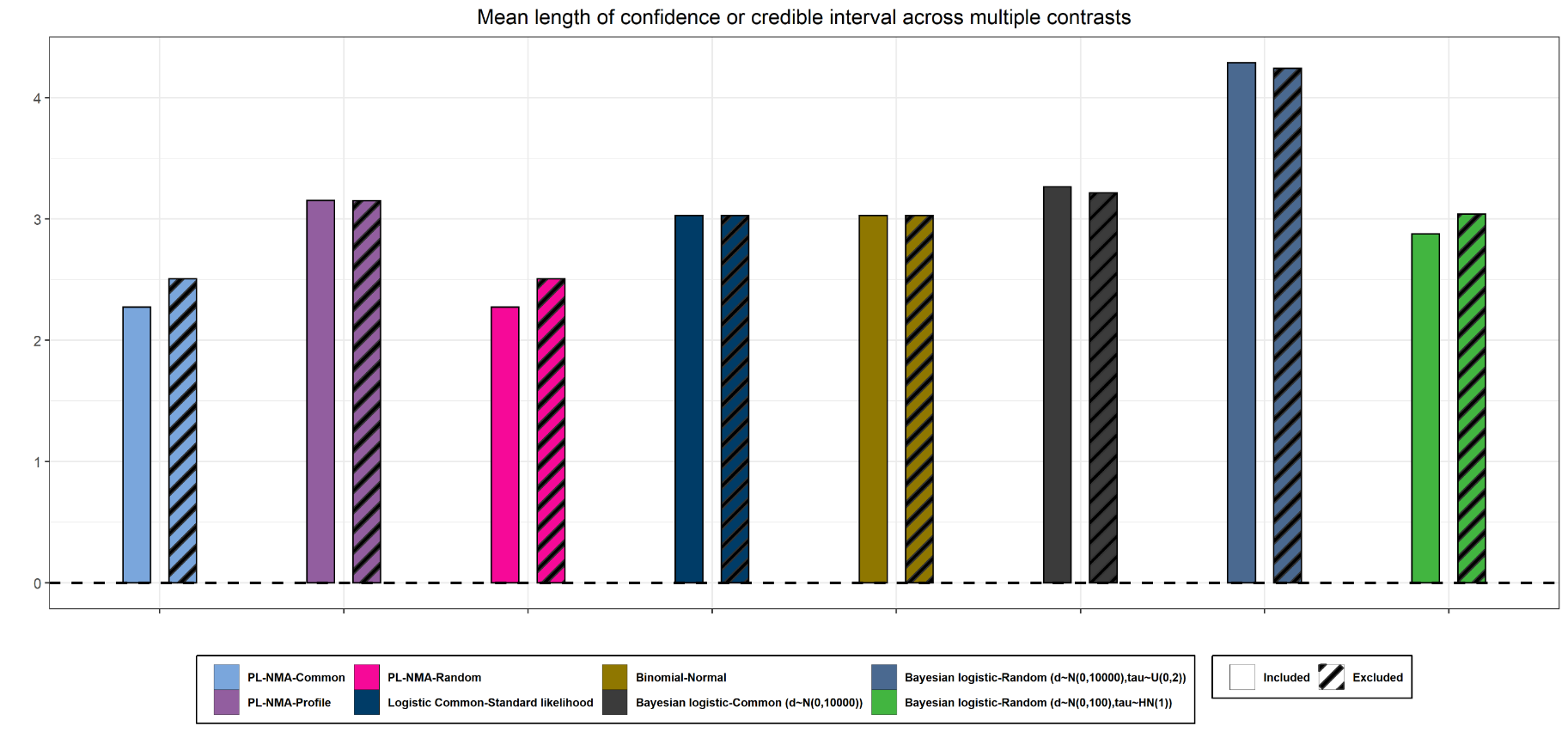


**S-Figure 8:** Mean length of confidence or credible interval across multiple contrasts for Scenario 33.

### Additional results for the clinical examples

#### Safety of inhaled medications for patients with chronic obstructive pulmonary disease

**S-Table 4:** Numerical results for the forest plot of the inhaled medications clinical example.

| **Model** | **ICS** | **LABA** | **LABA-ICS** | **TIO-HH** | **TIO-SMI** |
| --- | --- | --- | --- | --- | --- |
| **Mantel-Haenszel** | 1.03  [0.88,1.21] | 0.93  [0.79,1.08] | 0.79  [0.67,0.94] | 0.92  [0.81,1.05] | 1.52  [1.05,2.19] |
| **Non Central Hypergeometric** | 1.02  [0.88,1.19] | 0.94  [0.81,1.09] | 0.81  [0.69, 0.95] | 0.93  [0.82, 1.05] | 1.50  [1.05, 2.14] |
| **Penalized likelihood**  **(Wald type,**  **Profile likelihood)** | 1.03  [0.88, 1.21],  [0.88 , 1.21] | 0.93  [0.79, 1.09],  [0.79, 1.09] | 0.80  [0.67,0.94],  [0.67, 0.94] | 0.92  [0.81, 1.04],  [0.81, 1.04] | 1.50  [1.05, 2.16],  [1.05, 2.15] |
| **Logistic-Common Standard likelihood** | 1.03  [0.88, 1.21] | 0.93  [0.79, 1.09] | 0.79  [0.67, 0.94] | 0.92  [0.81, 1.04] | 1.51  [1.05, 2.17] |
| **Bayesian logistic-Common (d~N(0,10000))** | 1.03  [0.88, 1.21] | 0.93  [0.79, 1.09] | 0.79  [0.67, 0.94] | 0.92  [0.81, 1.04] | 1.52  [1.06, 2.20] |
| **Bayesian logistic-Random (d~N(0,10000),tau~U(0,2))** | 0.99  [0.77, 1.24] | 0.95  [0.76, 1.21] | 0.79  [0.62, 1.03] | 0.92  [0.75, 1.15] | 1.52  [1.00, 2.38] |
| **Bayesian logistic-Random (d~N(0,100),tau~HN(1))** | 0.99  [0.76, 1.24] | 0.95  [0.76, 1.21] | 0.79  [0.62, 1.02] | 0.92  [0.75, 1.15] | 1.54  [1.01, 2.38] |
| **Heterogeneity parameters**   - **PL-NMA:** $\hat{\varphi}=1$ - **Bayesian logistic-Random (d~N(0,10000), tau~U(0,2)):** $\hat{\tau}$=0.12 [0.01, 0.34] - **Bayesian logistic-Random (d~N(0,100), tau~HN(1)):** $\hat{\tau}$=0.12 [0, 0.35] | | | | | |

#### Safety of different drug classes for chronic plaque psoriasis

A two step procedure for the exclusion of zero event studies applies for MH-NMA and NCH-NMA^3^. At the first step all-zero event studies are excluded. After this step, the treatment design Anti-IL 23 vs. Placebo remains with studies that report zero events only for Placebo group. At a second step MH-NMA and NCH-NMA are searching within each treatment design for the treatment arms for which all studies in the design report zero events and remove those treatment arms from the design. As a result, the second step leads to the further exclusion of the Placebo arms in the design Anti-IL 23 vs Placebo; this treatment design then remains with only two single arm studies and thus it is completely excluded from the analysis. The same two stage procedure of exclusion applies to all treatment designs and leads to treatment designs Anti-IL 23 vs. Anti-IL 12/23 and Anti-IL 23 vs. Anti-TNF to be excluded from the analysis. All the treatment designs that involve the treatment Anti-IL 23 are needed to be removed and as a result the Anti-IL 23 nodes is discarded from the network.

**S-Table 5**: Numerical results for the forest plot of the psoriasis clinical example.

| **Model** | **Anti-IL 12/23** | **Anti-IL 17** | **Anti-IL 23** | **Anti-TNF** | **Apremilast** |
| --- | --- | --- | --- | --- | --- |
| **Mantel-Haenszel** | 1.23  [0.12, 12.50] | 0.97  [0.14, 6.72] | No results | 0.91  [0.14, 5.84] | 0.37  [0.02, 7.02] |
| **Non Central Hypereometric** | 1.28  [0.10, 16.17] | 1.11  [0.19, 6.43] | No results | 0.91  [0.14, 5.71] | 0.41  [0.02, 6.63] |
| **Penalized likelihood**  **(Wald type,**  **Profile likelihood)** | 1.30  [0.26, 6.62],  [0.11, 19.55] | 0.85  [0.29, 2.54],  [0.22, 4.23] | 2.54  [0.40, 16.09],  [0.12, 72.87) | 1.45  [0.46, 4.60],  [0.19, 13.23] | 0.43  [0.06, 2.91],  [0.05, 4.83] |
| **Logistic-Common Standard likelihood** | 1.34  [0.11, 15.98] | 0.96  [0.21, 4.29] | 5.72$\times$10^9^  [0, Inf)  (No results) | 1.60  [0.26, 9.68] | 0.41  [0.03, 6.64] |
| **Bayesian logistic-Common (d~N(0,10000))** | 1.37  [0.10, 21.23] | 1.08  [0.23, 6.18] | 1.87$\times$10^4^  [0.73, 1197$\times$10^15^]  (No results) | 1.80  [0.28, 14.90] | 0.40  [0.01, 15.04] |
| **Bayesian logistic-Random (d~N(0,10000),tau~U(0,2))** | 1.61  [0.08, 33.18] | 1.35  [0.19, 12.63] | 5.10$\times$10^5^  [0.94, 7.83$\times$10^15]^  (No results) | 2.32  [0.25, 28.98] | 0.38  [0.01, 23.49] |
| **Bayesian logistic-Random (d~N(0,100),tau~HN(1))** | 0.66  [0.06, 7.81] | 0.63  [0.12-2.85] | 1.03  [0.09, 16.80] | 0.74  [0.13, 4.23] | 0.28  [0.01, 7.09] |
| **Heterogeneity parameters**   - **PL-NMA:** $\hat{\varphi}=1$ - **Bayesian logistic-Random (d~N(0,10000), tau~U(0,2)):** $\hat{\tau}$=0.97 [0.05, 1.94] - **Bayesian logistic-Random (d~N(0,100), tau~HN(1)):** $\hat{\tau}$=0.56 [0.03, 1.61] | | | | | |

**References**

1. Efthimiou O, Debray TP, van Valkenhoef G, et al. GetReal in network meta‐analysis: a review of the methodology. *Res Synth Methods*. 2016;7(3):236-63.

2. Chaimani A, Caldwell DM, Li T, Higgins JP, Salanti G. Undertaking network meta-analyses. In: *Cochrane Handbook for Systematic Reviews of Interventions*. John Wiley & Sons, Ltd; 2019:285-320. doi:10.1002/9781119536604.ch11

3. Efthimiou O, Rücker G, Schwarzer G, Higgins JPT, Egger M, Salanti G. Network meta-analysis of rare events using the Mantel-Haenszel method. *Stat Med*. 2019;38(16):2992-3012.

4. Stijnen T, Hamza TH, Ozdemir P. Random effects meta-analysis of event outcome in the framework of the generalized linear mixed model with applications in sparse data. *Stat Med*. 2010;29(29):3046-67.

5. Simmonds MC, Higgins JP. A general framework for the use of logistic regression models in meta-analysis. *Stat Methods Med Res*. 2016;25(6):2858-2877.

6. Seide SE, Jensen K, Kieser M. A comparison of Bayesian and frequentist methods in random‐effects network meta‐analysis of binary data. *Res Synth Methods*. 2020;11(3):363-378.

7. Hu D, O’Connor AM, Wang C, Sargeant JM, Winder CB. How to conduct a bayesian network meta-analysis. *Front Vet Sci*. 2020;7.

8. Lu G, Ades AE. Assessing evidence inconsistency in mixed treatment comparisons. *J Am Stat Assoc*. 2006;101(474):447-459.

9. Greenland S. Generalized Mantel-Haenszel estimators for K 2 x J tables. *Biometrics*. 1989;45(1):183-191.

10. Gerta Rücker and Ulrike Krahn and Jochem König and Orestis Efthimiou and Guido Schwarzer. *Netmeta: Network Meta-Analysis Using Frequentist Methods*.; 2020. https://CRAN.R-project.org/package=netmeta
